# A Literature-Derived Muscle Wasting-Related Prognostic Signature Identifies ZBTB7C as a Candidate Associated with Tumor-Related Skeletal Muscle Alterations in Esophageal Cancer

**DOI:** 10.3390/cancers18213479

**Published:** 2026-10-28

**Authors:** Yan Li, Chang Xie, Rui Chen, Zhibing Ming, Haitao Huang, Shengguang Ding

**Affiliations:** 1 Cardiovascular Surgery Department, Nantong First People’s Hospital, Southeast University, Nantong 226001, China; 2113320228@stmail.ntu.edu.cn; 2 Vascular Surgery Department, Affiliated Nantong Clinical College of Nantong University, Nantong First People’s Hospital, Nantong 226001, China; xiechang2001@stmail.ntu.edu.cn (C.X.); 5300022@ntu.edu.cn (Z.M.); 3 Pathology Department, Nantong University, Nantong 226001, China; 2331310038@stmail.ntu.edu.cn; 4 Thoracic Surgery Department, Nantong First People’s Hospital, Southeast University, Nantong 226001, China

**Keywords:** esophageal cancer, skeletal muscle wasting, cancer cachexia, ZBTB7C, prognostic signature, esophageal squamous cell carcinoma, C2C12

## Abstract

**Background:** Skeletal muscle wasting is a frequent systemic complication of esophageal cancer and is associated with unfavorable clinical outcomes. However, the relationships among tumor-associated molecular characteristics, patient prognosis, and skeletal muscle alterations remain incompletely understood. This study aimed to identify prognostically relevant genes from a literature-curated muscle wasting-related gene set and investigate the potential association of ZBTB7C with tumor-related skeletal muscle alterations in esophageal squamous cell carcinoma (ESCC). **Methods:** Transcriptomic and survival data from the complete TCGA-ESCA cohort and the independent GSE53625 cohort were analyzed using a predefined set of genes implicated in muscle wasting-related biology. Differential expression analysis, consensus clustering, Cox regression, LASSO-Cox analysis, survival analysis, functional enrichment, and immune-related analyses were performed. A six-gene prognostic signature was constructed in the TCGA cohort and externally evaluated in GSE53625. ZBTB7C was subsequently investigated using human ESCC tissues and cell lines, Zbtb7c-overexpressing AKR cells, AKR-conditioned medium-treated C2C12 myotubes, and an immunocompetent AKR/C57BL/6 syngeneic tumor model. **Results:** A total of 32 literature-curated muscle wasting-related genes were retained for analysis. A six-gene prognostic signature comprising ZBTB7C, DDIT3, RNF157, AMOTL1, TTLL12, and PDLIM2 was associated with overall survival, although its discriminatory performance was limited in the external validation cohort. ZBTB7C expression was reduced in ESCC tissues and cell lines, and higher expression was associated with favorable survival. Zbtb7c overexpression reduced PCNA expression in AKR tumor cells. Conditioned medium from Zbtb7c-overexpressing AKR cells attenuated MuRF-1 and Atrogin-1 expression, partially restored AKT, FOXO3A, and P70S6K phosphorylation, and increased global protein synthesis in C2C12 myotubes. In vivo, Zbtb7c overexpression suppressed tumor growth and was accompanied by preservation of gastrocnemius muscle mass, attenuation of muscle protein homeostasis-related alterations, and a smaller decline in all-limb grip strength. **Conclusions:** These findings identify ZBTB7C as an exploratory candidate associated with esophageal cancer prognosis and tumor-related skeletal muscle alterations. However, its muscle-related effects cannot be fully distinguished from reduced tumor burden, and further mechanistic and clinical validation is required before its potential utility as a biomarker or therapeutic target can be established.

## Simple Summary

Skeletal muscle wasting is a common systemic complication of esophageal cancer and is associated with unfavorable clinical outcomes. In this study, we integrated transcriptomic and survival data from the TCGA-ESCA cohort with a literature-curated set of muscle wasting-related genes to develop a six-gene prognostic signature. Because the analyzed clinical datasets did not contain direct measurements of skeletal muscle mass or cachexia, the signature reflects the prognostic relevance of genes previously implicated in muscle wasting-related biology rather than a clinically validated muscle wasting phenotype. ZBTB7C was subsequently selected for experimental investigation. ZBTB7C expression was reduced in esophageal squamous cell carcinoma tissues and cell lines, whereas higher expression was associated with favorable survival. Zbtb7c overexpression reduced PCNA expression in AKR tumor cells and attenuated alterations in muscle protein homeostasis-related markers in C2C12 myotubes exposed to tumor-conditioned medium. In an immunocompetent syngeneic mouse model, Zbtb7c overexpression suppressed tumor growth and was accompanied by preservation of tumor-free body weight and gastrocnemius muscle mass. These findings suggest that ZBTB7C may be associated with tumor-related skeletal muscle alterations, although its underlying mechanisms and clinical relevance require further investigation.

## 1. Introduction

Esophageal cancer (EC) is one of the most aggressive gastrointestinal malignancies and remains associated with substantial morbidity and mortality worldwide [1,2]. Despite advances in surgery, chemoradiotherapy, targeted therapy, and immunotherapy, the prognosis of patients with advanced disease remains unsatisfactory. In addition to local tumor progression, many patients experience progressive weight loss, nutritional deterioration, and loss of skeletal muscle mass during the course of disease [3,4]. Skeletal muscle wasting is particularly relevant in EC because it may impair physical performance, reduce treatment tolerance, increase postoperative complications, and adversely affect long-term outcomes [5,6]. Therefore, elucidating the biological factors associated with skeletal muscle loss may provide additional insight into the systemic manifestations of EC.

Skeletal muscle wasting refers to the progressive loss of skeletal muscle mass and is a major component of cancer cachexia, a multifactorial syndrome involving systemic metabolic disturbances and impaired nutritional and functional status. However, skeletal muscle wasting and cancer cachexia are not interchangeable concepts, as muscle loss may occur without sufficient evidence to establish a clinical diagnosis of cachexia. In the present study, the term “tumor-associated muscle wasting” is used to describe skeletal muscle loss occurring under tumor-bearing conditions, whereas changes in muscle atrophy-associated proteins and protein synthesis-related signaling are described as alterations in skeletal muscle protein homeostasis [7,8]. Tumor-derived factors, systemic inflammation, metabolic disturbances, and impaired anabolic signaling collectively contribute to this process [9]. At the molecular level, activation of catabolic pathways promotes the expression of muscle-specific ubiquitin ligases, including muscle RING finger 1 (MuRF-1/TRIM63) and muscle atrophy F-box protein (Atrogin-1/FBXO32), whereas suppression of anabolic signaling further compromises protein synthesis [7]. The AKT–FOXO3A axis and protein synthesis-related signaling are important regulators of skeletal muscle protein homeostasis and are frequently disturbed during cancer-associated muscle wasting [10,11]. However, the tumor-intrinsic molecular factors that contribute to these systemic skeletal muscle alterations remain incompletely understood.

Most previous studies in EC have focused on the clinical significance of sarcopenia, body composition, nutritional status, and skeletal muscle index [12,13]. Although these studies have established the prognostic relevance of muscle depletion [14], the molecular relationship between tumor biology and skeletal muscle wasting has received comparatively less attention. Identifying tumor-related genes that are simultaneously associated with prognosis and muscle wasting may therefore help bridge tumor-intrinsic molecular alterations with systemic host responses and provide candidate biomarkers for further mechanistic investigation. For example, an ESCC-specific clinical study involving 85 patients receiving neoadjuvant immunotherapy plus chemotherapy demonstrated that changes in CT-derived skeletal muscle index were associated with pathological treatment response and postoperative complications [15]. These findings highlight the clinical importance of longitudinal skeletal muscle assessment in ESCC. However, the relationships between such clinically measured muscle phenotypes and tumor-intrinsic molecular characteristics remain insufficiently understood. Accordingly, studies based on literature-curated muscle wasting-related genes should be distinguished from investigations in which muscle depletion or cachexia has been directly quantified in individual patients.

Zinc finger and BTB domain-containing protein 7C (ZBTB7C) is a transcriptional regulator belonging to the BTB/POZ zinc-finger protein family [16]. Previous studies have suggested that ZBTB7C participates in tumor-associated transcriptional regulation and displays altered expression across different malignancies [16,17,18]. However, its biological significance in esophageal cancer remains insufficiently characterized, and whether tumor-cell ZBTB7C is associated with cancer-related skeletal muscle wasting has not been established [18,19]. These observations prompted us to investigate ZBTB7C as a potential molecular link between esophageal tumor biology and systemic skeletal muscle alterations.

In the present study, we integrated transcriptomic and clinical data from the complete TCGA-ESCA cohort [20] with a predefined muscle wasting-related gene set to identify prognostically relevant genes in esophageal cancer. Differential expression analysis, protein–protein interaction analysis, consensus clustering, Cox regression, least absolute shrinkage and selection operator analysis, functional enrichment, and immune-related analyses were performed, leading to the establishment of a six-gene prognostic signature comprising ZBTB7C, DDIT3, RNF157, AMOTL1, TTLL12, and PDLIM2. ZBTB7C was subsequently selected as an exploratory candidate for experimental investigation based on its prognostic association, differential expression, transcriptional regulatory function, and the limited understanding of its involvement in tumor-associated skeletal muscle alterations. Its biological relevance to tumor-associated skeletal muscle wasting was further evaluated using Zbtb7c-overexpressing AKR cells, AKR-conditioned medium-treated C2C12 myotubes, and an immunocompetent AKR/C57BL/6 syngeneic tumor model. Through this integrated bioinformatic and experimental approach, we aimed to characterize the potential role of ZBTB7C in linking esophageal tumor biology with skeletal muscle wasting.

## 2. Materials and Methods

### 2.1. Data Acquisition and Preprocessing

Transcriptomic profiles and corresponding clinical information were obtained from The Cancer Genome Atlas Esophageal Carcinoma (TCGA-ESCA) cohort and used as the training cohort. The complete TCGA-ESCA cohort included both esophageal squamous cell carcinoma (ESCC) and esophageal adenocarcinoma (EAC), whereas the independent GSE53625 cohort comprised ESCC patients. The complete TCGA-ESCA cohort was retained for the original discovery and prognostic model-development analyses. No histology-specific model development was performed; therefore, prognostic associations derived from the mixed TCGA-ESCA cohort were not assumed to be directly generalizable to individual histological subtypes. An independent external validation cohort was obtained from the Gene Expression Omnibus (GEO) database under accession number GSE53625. For TCGA-ESCA, gene expression values reported as fragments per kilobase of transcript per million mapped reads (FPKM) were converted to transcripts per million (TPM) for subsequent bioinformatic analyses. Samples with incomplete survival follow-up information or missing survival status were excluded. A predefined set of muscle wasting-related genes was manually curated from previously published peer-reviewed studies [21,22,23,24,25,26,27,28,29,30,31,32,33,34,35,36,37,38,39,40,41,42,43,44,45,46,47,48,49,50,51,52], followed by gene-symbol standardization and removal of duplicate entries. Detailed information on the included genes and their supporting references is provided in Supplementary Table S1. After intersecting the curated gene set with the available transcriptomic data, 32 muscle wasting-related genes were retained for downstream analyses. Importantly, the muscle wasting-related gene set was curated from previously published literature rather than derived from directly measured skeletal muscle phenotypes in the analyzed patients. Neither TCGA-ESCA nor GSE53625 contained individual-level measurements of skeletal muscle mass, sarcopenia, cachexia status, or longitudinal body-composition changes suitable for phenotype-based validation. Accordingly, the designation “muscle wasting-related” refers to the biological background of the candidate genes, and the subsequent prognostic analyses were based on overall survival rather than clinically documented muscle wasting. 

### 2.2. Differential Expression and Protein–Protein Interaction Analyses

Differential expression analysis of 32 muscle wasting-related genes between ESCA tumor and adjacent normal tissues was performed using the limma R package. Genes with |log2FC| > 1 and FDR < 0.05 were defined as statistically significant differentially expressed genes (DEGs). The Benjamini–Hochberg method was applied for multiple testing correction. Expression patterns of candidate genes were visualized via boxplots and heatmaps. The protein–protein interaction (PPI) network of DEGs was constructed using the online STRING database (version 11.5). Only interaction pairs with a combined confidence score > 0.4 were retained. The PPI network was imported into Cytoscape (v3.9.1) for topological visualization. Pearson correlation analysis was further conducted to evaluate the coordinated expression relationships among candidate muscle wasting-related genes.

### 2.3. Consensus Clustering and Molecular Subtyping

Unsupervised consensus clustering was performed using the ConsensusClusterPlus R package based on the expression matrix of 32 muscle wasting-related genes. The clustering procedure was repeated 1000 iterations with 80% sample resampling, Pearson correlation distance, and a maximum cluster number of 9. The optimal subtype number was comprehensively determined according to the consensus matrix, cumulative distribution function (CDF) curve, and relative delta area change. All ESCA samples were finally classified into three distinct molecular subtypes (C1, C2, C3). Kaplan–Meier survival analysis combined with the log-rank test was applied to compare overall survival differences among the three subtypes, with *p* < 0.05 considered statistically significant.

### 2.4. Construction of the Muscle Wasting-Related Prognostic Signature

Univariate Cox proportional hazards regression analysis was first performed in the TCGA training cohort to identify muscle wasting-related genes associated with overall survival, and genes with *p* < 0.05 were retained as candidate prognostic genes. Before model construction, gene-level Z-score normalization was performed within each cohort to reduce the influence of differences in expression scale and detection platform.

Least absolute shrinkage and selection operator (LASSO)-Cox regression analysis was subsequently performed in the TCGA training cohort using the glmnet R package to reduce overfitting and determine the final prognostic signature. Ten-fold cross-validation was used to select the optimal penalty parameter (λ) corresponding to the minimum partial likelihood deviance, and genes with non-zero coefficients at the optimal λ were retained in the final model. The risk score for each patient was calculated according to the following formula: Risk score = Σ (Z-score-normalized gene expression × corresponding LASSO regression coefficient). The regression coefficients derived from the TCGA training cohort were fixed and subsequently applied to the GSE53625 validation cohort to calculate individual risk scores. Within each cohort, gene expression values were standardized separately using gene-level Z-score normalization, and patients were stratified into high- and low-risk groups according to the median risk score of the corresponding cohort. Therefore, although the regression coefficients were retained across cohorts, the external evaluation did not assess the transportability of a fully prespecified risk classification threshold. Univariate and multivariate Cox proportional hazards regression analyses were subsequently performed to evaluate the prognostic significance of the risk score together with available clinicopathological variables.

Within each cohort, patients were independently stratified into high- and low-risk groups according to the median risk score of the corresponding cohort. Although the LASSO regression coefficients derived from the TCGA training cohort were fixed and applied to GSE53625, gene expression values were standardized separately within each cohort, and cohort-specific median cutoffs were used for risk stratification. Therefore, the external analysis evaluated the prognostic association of the risk score within the independent cohort rather than the transportability of a fully prespecified risk classification threshold. Univariate and multivariate Cox proportional hazards regression analyses were subsequently performed to evaluate the prognostic significance of the risk score together with available clinicopathological variables.

### 2.5. Evaluation of the Prognostic Model

Multiple approaches were adopted to systematically validate the predictive performance of the six-gene prognostic signature. The risk score distribution, survival status distribution, and gene expression ranking of all patients were visualized in both TCGA training and GSE53625 validation cohorts. Kaplan–Meier survival curves with log-rank tests were used to compare survival differences between high- and low-risk groups. Time-dependent receiver operating characteristic (time-ROC) curves at 1, 3, and 5-year overall survival were plotted using the timeROC R package with the Aalen weighting method to calculate AUC values. t-distributed stochastic neighbor embedding (t-SNE) dimensionality reduction analysis was performed via the Rtsne package to verify the discrete distribution patterns of high-risk and low-risk samples based on the signature gene expression profiles.

### 2.6. Expression Characteristics of the Six Prognostic Genes

The expression differences in six signature genes (ZBTB7C, DDIT3, RNF157, AMOTL1, TTLL12, PDLIM2) between high- and low-risk subgroups were systematically compared in both TCGA and GSE53625 cohorts. A comprehensive prognostic heatmap integrating signature gene expression profiles and clinical phenotypic characteristics was plotted using the pheatmap package. Chi-square tests were applied to evaluate the correlation between risk grouping and clinical features, with significance levels marked as * *p* < 0.05, ** *p* < 0.01, *** *p* < 0.001.

### 2.7. Functional Enrichment Analysis

DEGs between high-risk and low-risk groups were screened via Wilcoxon rank-sum test with thresholds of |logFC| > 1 and FDR < 0.05. Gene Ontology (GO) and Kyoto Encyclopedia of Genes and Genomes (KEGG) enrichment analyses were performed using the clusterProfiler R package (v4.6.2) and human gene annotation database org.Hs.eg.db. Enrichment terms with *p* < 0.05 and q-value < 0.05 were considered statistically significant. Bar plots and bubble plots were generated to visualize the enrichment results of biological processes, cellular components, molecular functions, and enriched signaling pathways.

### 2.8. ssGSEA-Based Immune Analysis

Single-sample gene set enrichment analysis (ssGSEA) was conducted using the GSVA and GSEABase R packages to quantify the relative enrichment levels of immune cell infiltration and immune functional pathways in individual samples. Analysis parameters were set as: method = “ssgsea”, kcdf = “Gaussian”, abs.ranking = TRUE. All ssGSEA scores were normalized to a range of [0, 1] via min–max standardization. Immune-related gene sets were derived from the MSigDB C7 immunologic signature dataset. Differences in immune infiltration and immune functional scores between high- and low-risk groups were compared in both TCGA training and GSE53625 validation cohorts.

### 2.9. ZBTB7C Single-Gene Analysis

ZBTB7C was selected from the six-gene prognostic signature for exploratory investigation based on a combination of considerations, including its significant association with overall survival, differential expression between esophageal tumor and non-tumor tissues, its known function as a transcriptional regulator, and the limited understanding of its potential involvement in tumor-associated skeletal muscle alterations. This selection represented a biologically informed prioritization rather than a formal quantitative ranking of the six prognostic genes. In particular, ZBTB7C was not selected on the basis of having the strongest prognostic effect size or the highest network connectivity. Expression differences of ZBTB7C between tumor and normal samples were verified. Patients were stratified into ZBTB7C-high and ZBTB7C-low subgroups according to the median expression value, and Kaplan–Meier survival analysis was performed. DEGs between the two ZBTB7C expression subgroups were identified with |log2FC| > 1 and FDR < 0.05 and visualized by volcano plots. The FDR-adjusted *p*-values were used to account for multiple testing during differential gene expression analysis. GO and KEGG enrichment analyses were performed to explore potential biological functions of ZBTB7C. In addition, ssGSEA was used to quantitatively compare differences in immune-related scores, including CCR activity, HLA expression, parainflammation, and Treg infiltration, between ZBTB7C-high and ZBTB7C-low subgroups.

### 2.10. Human Tissue Samples

A total of 28 paired esophageal squamous cell carcinoma (ESCC) tissues and corresponding adjacent non-tumor tissues were obtained from patients who underwent surgical resection at Nantong First People’s Hospital. Each pair consisted of tumor and adjacent non-tumor tissue specimens obtained from the same patient, resulting in 28 independent patient-level biological pairs. All specimens were obtained from residual surgical materials remaining after routine pathological examination and were not collected through additional invasive procedures for research purposes. Fresh tissue specimens were stored at −80 °C until protein extraction. The paired tissue cohort was used to evaluate differences in ZBTB7C protein expression between ESCC tumors and corresponding adjacent non-tumor tissues. ZBTB7C protein expression was measured by Western blotting, with GAPDH used as the loading control. The normalized protein expression levels were compared within matched tissue pairs. This study was approved by the Ethics Committee of Nantong First People’s Hospital on 1 September 2026 (Approval No. 2026-KT356-01). The requirement for written informed consent was waived by the ethics committee because the study involved retrospective use of residual surgical specimens.

### 2.11. Cell Lines and Cell Culture

The immortalized human esophageal epithelial cell line Het-1A was obtained from the American Type Culture Collection (ATCC), whereas the human esophageal squamous cell carcinoma (ESCC) cell lines TE-1, KYSE30, KYSE150, and KYSE510 were obtained from the Cell Bank of the Chinese Academy of Sciences (Shanghai, China). HCE-4 cells (Cellosaurus accession: CVCL_1271) were also used as a human ESCC cell line. Het-1A cells were cultured in bronchial epithelial growth medium (BEGM) supplemented with 10% fetal bovine serum (FBS). TE-1, KYSE150, and KYSE510 cells were cultured in RPMI-1640 medium, HCE-4 cells were cultured in Dulbecco’s modified Eagle’s medium (DMEM), and KYSE30 cells were cultured in a 1:1 mixture of RPMI-1640 and Ham’s F-12 medium. Unless otherwise indicated, the culture media were supplemented with 10% FBS and 1% penicillin–streptomycin. AKR cells were obtained from Shanghai Zhong Qiao Xin Zhou Biotechnology Co., Ltd. (Shanghai, China) and routinely cultured in DMEM supplemented with 10% FBS and 1% penicillin–streptomycin; cells at passages 1–5 were used for the experiments. C2C12 mouse myoblasts were obtained from the Cell Bank of Type Culture Collection, Chinese Academy of Sciences (Shanghai, China) and routinely maintained in DMEM supplemented with 10% FBS and 1% penicillin–streptomycin. All cells were maintained at 37 °C in a humidified atmosphere containing 5% CO_2_. For subsequent experiments, C2C12 myogenic differentiation was performed as described in Section 2.13. ZBTB7C protein expression in Het-1A, TE-1, HCE-4, KYSE30, KYSE150, and KYSE510 cells was evaluated by Western blotting.

### 2.12. Zbtb7c Overexpression in AKR Cells

A lentiviral vector encoding the full-length mouse Zbtb7c sequence (Zbtb7c-OE) and the corresponding empty-vector control (Vector) were designed, constructed, and packaged by GenePharma (Suzhou, China). The lentiviral titre provided by the manufacturer was >1 × 10^9^ TU/mL. For transduction, AKR cells were seeded in 24-well plates at 2 × 10^4^ cells/well and infected with Zbtb7c-OE or Vector lentivirus at a multiplicity of infection (MOI) of 20 in the presence of polybrene (5 μg/mL). At 24 h after infection, the culture medium was replaced with fresh complete medium, and stable transductants were selected with puromycin (2 μg/mL) for 1 week. ZBTB7C overexpression was confirmed by Western blotting, and PCNA protein expression was subsequently examined in AKR-Vector and AKR-Zbtb7c-OE cells. GAPDH was used as the loading control.

### 2.13. AKR-Conditioned Medium Treatment of C2C12 Myotubes

For the preparation of AKR-conditioned medium (AKR-CM), AKR-Vector and AKR-Zbtb7c-OE cells were cultured to approximately 70–80% confluence. The routine culture medium was then replaced with serum- and antibiotic-free DMEM, and the cells were incubated for an additional 48 h. Conditioned supernatants were collected and centrifuged at 1000× *g* for 5 min at room temperature to remove detached cells and cellular debris. The clarified conditioned media were stored at 4 °C and used within 24 h. The same conditioning duration and medium composition were used for both AKR-Vector and AKR-Zbtb7c-OE cells. However, conditioned media were not normalized to viable tumor-cell number or cellular protein content at the end of the collection period, and quantitative cell viability measurements were not performed during conditioned-medium collection. For myogenic differentiation, C2C12 cells were cultured in 100-mm dishes to approximately 80% confluence, after which the growth medium was replaced with differentiation medium consisting of DMEM supplemented with 2% horse serum. Cells were differentiated for 6 days to allow the formation of mature multinucleated myotubes. Differentiated C2C12 myotubes were assigned to the Control, Vector-CM, and Zbtb7c-OE-CM groups. For the conditioned-medium groups, clarified AKR-Vector or AKR-Zbtb7c-OE conditioned supernatant was mixed at a 1:1 ratio with freshly prepared DMEM containing 4% horse serum, resulting in treatment media containing 50% conditioned supernatant and a final horse serum concentration of 2%. The Control group received a corresponding medium mixture consisting of 50% serum-free DMEM and 50% freshly prepared DMEM containing 4% horse serum, maintaining a final horse serum concentration of 2% without exposure to AKR-derived conditioned factors. C2C12 myotubes were treated for 48 h, with freshly prepared treatment medium replaced every 24 h. Cells were subsequently harvested for Western blotting and SUnSET analyses.

### 2.14. Assessment of Protein Synthesis in C2C12 Myotubes Using the SUnSET Method

Global protein synthesis in C2C12 myotubes was assessed using the surface sensing of translation (SUnSET) method based on puromycin incorporation into newly synthesized polypeptides. Following 48 h of treatment in the Control, Vector-CM, and Zbtb7c-OE-CM groups, puromycin was added directly to the corresponding culture medium at a final concentration of 1 μM. Cells were incubated for an additional 30 min and subsequently harvested for protein extraction. Puromycin incorporation was detected by Western blotting using an anti-puromycin antibody, as described in Section 2.15. The integrated density of puromycin-positive bands across the entire lane was quantified using ImageJ software (National Institutes of Health, Bethesda, MD, USA). The total puromycin signal was normalized to the corresponding GAPDH signal for each sample. The resulting normalized values were used as a relative indicator of global protein synthesis in C2C12 myotubes.

### 2.15. Western Blot Analysis

Total protein was extracted from human tissues, esophageal cell lines, AKR cells, C2C12 myotubes, tumor tissues, and gastrocnemius muscle tissues using radioimmunoprecipitation assay (RIPA) lysis buffer, and protein concentrations were determined using a bicinchoninic acid (BCA) assay. Equal amounts of protein (50 μg per lane) were separated by 10% sodium dodecyl sulfate–polyacrylamide gel electrophoresis (SDS–PAGE) and transferred onto polyvinylidene difluoride (PVDF) membranes. Membranes were blocked with 5% non-fat milk for 2 h at room temperature and subsequently incubated overnight at 4 °C with the corresponding primary antibodies. ZBTB7C expression was examined in human tissues, esophageal cell lines, AKR cells, and tumor tissues, whereas PCNA was evaluated in AKR cells and tumor tissues. MuRF-1, Atrogin-1, p-AKT, total AKT, p-FOXO3A (Ser253), total FOXO3A, p-P70S6K, and total P70S6K were analyzed in C2C12 myotubes and gastrocnemius muscle tissues. FOXO3A phosphorylation was quantified as the ratio of p-FOXO3A (Ser253) to total FOXO3A. This ratio was used to evaluate changes in phosphorylation at the specified regulatory site rather than as a direct measurement of FOXO3A transcriptional activity. After washing, the membranes were incubated with the appropriate secondary antibodies for 2 h at room temperature. Protein bands were visualized using enhanced chemiluminescence (ECL) and quantified by densitometric analysis. For the analysis of human ESCC tissue specimens, ZBTB7C band intensities were normalized to the corresponding GAPDH band intensities in each sample. The resulting normalized expression values were used for paired comparisons between tumor and adjacent non-tumor tissues from the same patient. GAPDH was used as the loading control for the quantification of total protein expression and puromycin incorporation. For phosphorylation analysis, the band intensities of p-AKT, p-FOXO3A, and p-P70S6K were normalized to those of their corresponding total proteins (AKT, FOXO3A, and P70S6K, respectively). The p-AKT/AKT, p-FOXO3A/FOXO3A, and p-P70S6K/P70S6K ratios were calculated for each sample and used for quantitative comparisons among experimental groups. These ratios were used to assess relative phosphorylation changes rather than absolute kinase activity. Detailed information on the antibodies used for Western blotting is provided in Supplementary Table S2.

### 2.16. Animal Experimental Design

Specific pathogen-free (SPF) male C57BL/6 mice, 6 weeks of age and weighing 20–22 g, were obtained from Nantong University. After a 7-day acclimatization period, mice were randomly assigned to three groups with four mice per group: Control, Vector, and Zbtb7c-OE. On Day 0, mice in the Vector and Zbtb7c-OE groups were subcutaneously inoculated with AKR-Vector or AKR-Zbtb7c-OE cells, respectively, at a dose of 2 × 10^6^ cells in 100 μL per mouse, whereas mice in the Control group did not receive tumor-cell inoculation. The experiment was continued for 28 days. On Day 28, mice were euthanized according to the approved ethical procedures, and tumors and gastrocnemius muscles were collected for subsequent analyses. All animals were maintained under SPF conditions throughout the experiment. All animal procedures were approved by the Animal Ethics Committee of Nantong University on 1 September 2026 (Approval No. P20260901-066) and were performed in accordance with institutional guidelines for the care and use of laboratory animals. The animal experiments were conducted as an exploratory in vivo investigation, with four mice included in each group. No formal prospective sample-size calculation was performed. Given the limited sample size, the findings were interpreted as preliminary experimental evidence requiring confirmation in larger, adequately powered cohorts.

### 2.17. Evaluation of Tumor Burden, Body Weight, Gastrocnemius Muscle Mass, and Grip Strength

Body weight was recorded at the beginning of the experiment and at the experimental endpoint. To minimize the influence of tumor mass on body weight assessment, tumor-free body weight in tumor-bearing mice was calculated as follows: tumor-free body weight = terminal body weight − excised tumor weight. The percentage change in tumor-free body weight was calculated using the formula: tumor-free body weight change (%) = (terminal tumor-free body weight − initial body weight)/initial body weight × 100%. At the experimental endpoint, tumors and gastrocnemius muscles were excised and photographed. Tumor weight was measured in the Vector and Zbtb7c-OE groups, whereas right gastrocnemius muscle weight was determined in the Control, Vector, and Zbtb7c-OE groups. Tumor tissues were subsequently subjected to Western blotting to evaluate ZBTB7C and PCNA protein expression. All-limb grip strength was assessed on experimental days 0, 7, 14, 21, and 28 using a grip strength meter (KW-ZL-2; Nanjing Calvin Biotechnology Co., Ltd., Nanjing, China) equipped with a metal grid. During testing, each mouse was placed on the grid and allowed to grasp it firmly with all four limbs. The mouse was then gently held by the tail and pulled horizontally backward at a steady speed until all four limbs completely released the grid. The maximal force generated immediately before release was recorded in grams (g). Each mouse underwent three consecutive measurements at each time point, with an interval of at least 30 s between measurements to minimize fatigue, and the mean of the three measurements was used for statistical analysis. Grip strength was monitored longitudinally throughout the experimental period. The percentage change in all-limb grip strength from baseline to the experimental endpoint was calculated as follows: change in all-limb grip strength (%) = (grip strength on day 28 − grip strength on day 0)/grip strength on day 0 × 100%. The same testing procedure was applied at all time points.

### 2.18. Molecular Analysis of Gastrocnemius Muscle

Gastrocnemius muscle tissues from the Control, Vector, and Zbtb7c-OE groups were subjected to Western blot analysis to determine the expression of MuRF-1, Atrogin-1, p-AKT, AKT, p-FOXO3A, FOXO3A, p-P70S6K, and P70S6K. GAPDH was used as the loading control, and the p-AKT/AKT, p-FOXO3A/FOXO3A, and p-P70S6K/P70S6K ratios were calculated to evaluate changes in signaling associated with skeletal muscle protein homeostasis.

### 2.19. Assessment of Skeletal Muscle Protein Synthesis In Vivo Using the SUnSET Method

Global skeletal muscle protein synthesis in mice was assessed using the surface sensing of translation (SUnSET) method. At the experimental endpoint, mice were intraperitoneally injected with puromycin at a dose of 0.04 μmol/g body weight (Yeasen Biotechnology, Shanghai, China; Cat. No. 60209ES60). Thirty minutes after puromycin administration, the mice were euthanized, and the gastrocnemius muscles were rapidly harvested for protein extraction. Puromycin incorporation into newly synthesized polypeptides was detected by Western blotting using the anti-puromycin antibody described in Section 2.15. The puromycin signal was quantified using ImageJ software by measuring the integrated density of all puromycin-positive bands across the entire lane. The total puromycin signal for each sample was normalized to the corresponding GAPDH signal, and the normalized puromycin incorporation signal was used as a relative indicator of global protein synthesis in gastrocnemius muscle during the puromycin-labeling period, rather than as an absolute measurement of protein synthesis rate.

### 2.20. Statistical Analysis

Statistical analyses and data visualization for the experimental studies were performed using GraphPad Prism version 9.3.1 (GraphPad Software, San Diego, CA, USA), whereas bioinformatics analyses and related statistical calculations were conducted using R software (v4.2.1). Unless otherwise indicated, data are presented as the mean ± standard deviation (SD). For the animal experiments, each individual mouse was considered an independent biological replicate (*n* = 4 mice per group). For in vitro experiments, each independently performed experiment was considered an independent biological replicate, and experiments were independently repeated at least three times. Technical measurements obtained within the same independent experiment were not treated as separate biological replicates. Data normality was assessed using the Shapiro–Wilk test. For comparisons between two independent groups, normally distributed data were analyzed using a two-tailed unpaired Student’s *t*-test, with Welch’s correction applied when the assumption of equal variances was not met, whereas non-normally distributed data were analyzed using the Mann–Whitney U test. For paired ESCC tumor and adjacent non-tumor tissues, normally distributed paired data were analyzed using a two-tailed paired Student’s *t*-test, whereas non-normally distributed paired data were analyzed using the Wilcoxon matched-pairs signed-rank test. For comparisons among three independent groups, normally distributed data were analyzed using ordinary one-way analysis of variance (ANOVA) followed by Dunnett’s multiple comparisons test for predefined comparisons. Non-normally distributed data were analyzed using the Kruskal–Wallis test followed by an appropriate multiple-comparisons procedure. Adjusted *p*-values from the applicable post hoc comparisons were used to assess statistical significance. For the bioinformatic analyses, differential expression testing was performed using the methods described in Section 2.2, Section 2.7, and Section 2.9. The Benjamini–Hochberg procedure was used to control the false discovery rate (FDR), and genes meeting the specified fold-change and FDR thresholds were considered differentially expressed. GO and KEGG enrichment analyses were evaluated using the reported *p*-value and q-value criteria. Kaplan–Meier survival analysis with the log-rank test was used to compare survival distributions, and univariate and multivariate Cox proportional hazards regression analyses were used to assess prognostic associations. Time-dependent receiver operating characteristic curves were generated to evaluate discrimination at the specified follow-up times. All statistical tests were two-sided, and *p* < 0.05 was considered statistically significant unless otherwise specified. Statistical significance in the figures was indicated as follows: * *p*< 0.05, ** *p* < 0.01, *** *p* < 0.001, and **** *p* < 0.0001.

## 3. Results

### 3.1. Identification of Muscle Wasting-Related Genes and Molecular Subtypes in Esophageal Cancer

**Figure 1:**
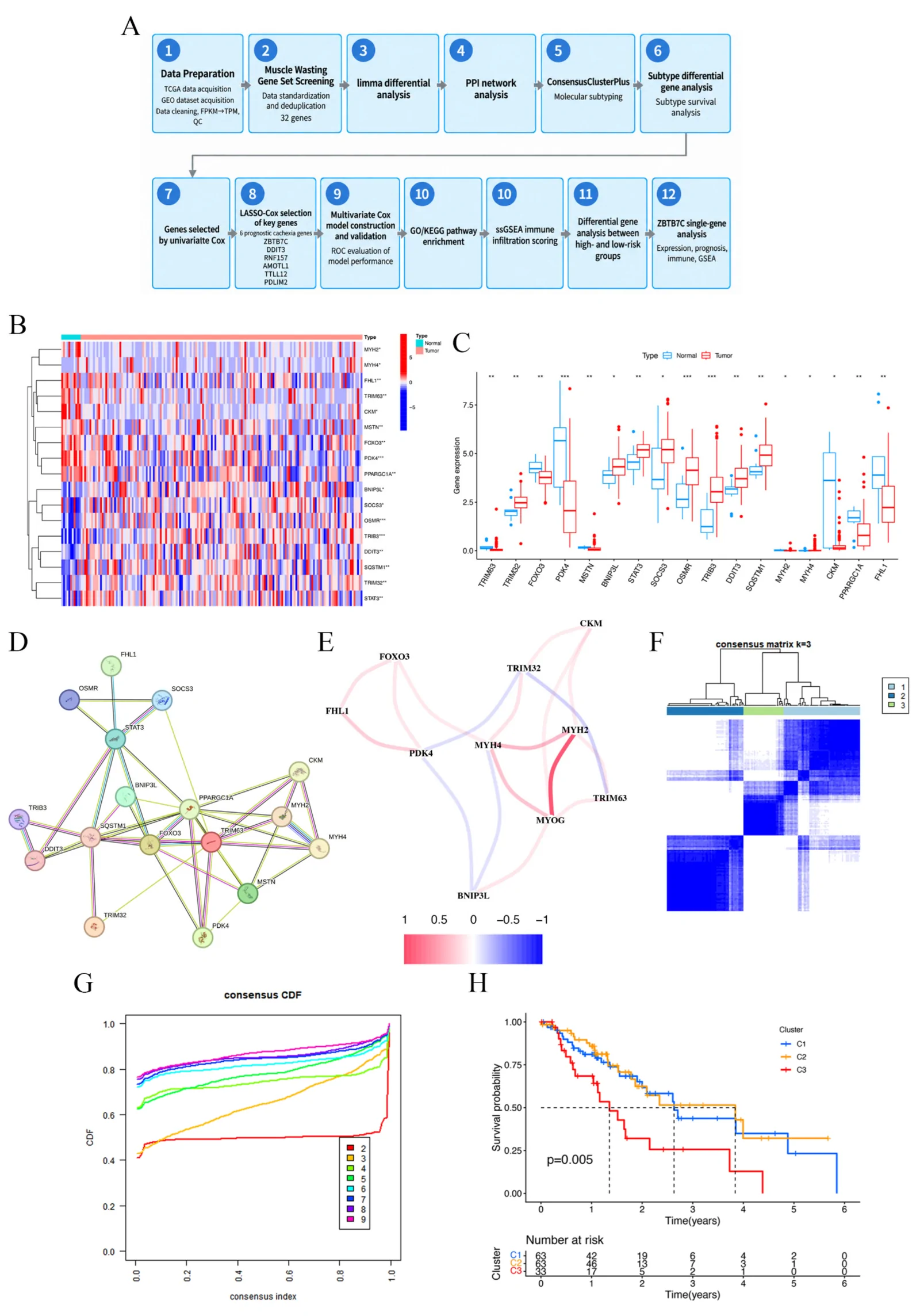
Identification of muscle wasting-related genes and molecular subtypes in esophageal cancer. (**A**) Overall workflow of the bioinformatic analyses. (**B**) Heatmap showing the expression patterns of muscle wasting-related genes in tumor and normal samples. (**C**) Boxplots comparing the expression levels of muscle wasting-related genes between tumor and normal samples. (**D**) Protein–protein interaction network of muscle wasting-related genes. (**E**) Correlation network showing relationships among muscle wasting-related genes; red and blue indicate positive and negative correlations, respectively. (**F**) Consensus clustering matrix for k = 3. (**G**) Consensus cumulative distribution function curves for different values of k. (**H**) Kaplan–Meier overall survival curves for the three molecular subtypes (C1, C2, and C3). The log-rank *p*-value is shown in the figure. * *p* < 0.05, ** *p* < 0.01, *** *p* < 0.001.

The overall study workflow is summarized in Figure 1A. Transcriptomic and clinical data from the complete TCGA-ESCA cohort, comprising both ESCC and EAC, were integrated with a literature-curated muscle wasting-related gene set. After data standardization and deduplication, 32 candidate genes were retained for subsequent analyses. Their expression patterns differed between esophageal tumor and non-tumor samples, with multiple genes showing significant dysregulation (Figure 1B,C). Protein–protein interaction and correlation analyses further demonstrated extensive functional connectivity among these genes, suggesting coordinated involvement in muscle- and tumor-associated biological processes (Figure 1D,E). The 32 candidate genes were derived from previously published studies of skeletal muscle wasting-related biological processes. Because direct skeletal muscle phenotypes were unavailable in the analyzed clinical cohorts, the observed expression differences and subsequent molecular classifications should be interpreted as tumor transcriptomic characteristics associated with a literature-curated gene set, rather than as evidence of clinically documented skeletal muscle wasting.

Unsupervised consensus clustering was subsequently performed according to the expression profiles of the muscle wasting-related genes. The consensus matrix and cumulative distribution function supported an optimal classification into three molecular subtypes (Figure 1F,G). Kaplan–Meier analysis revealed a significant difference in overall survival among the three clusters (log-rank *p* = 0.005; Figure 1H), indicating that muscle wasting-related transcriptional characteristics were associated with clinically distinct EC phenotypes.

### 3.2. Construction of a Six-Gene Muscle Wasting-Related Prognostic Signature

**Figure 2:**
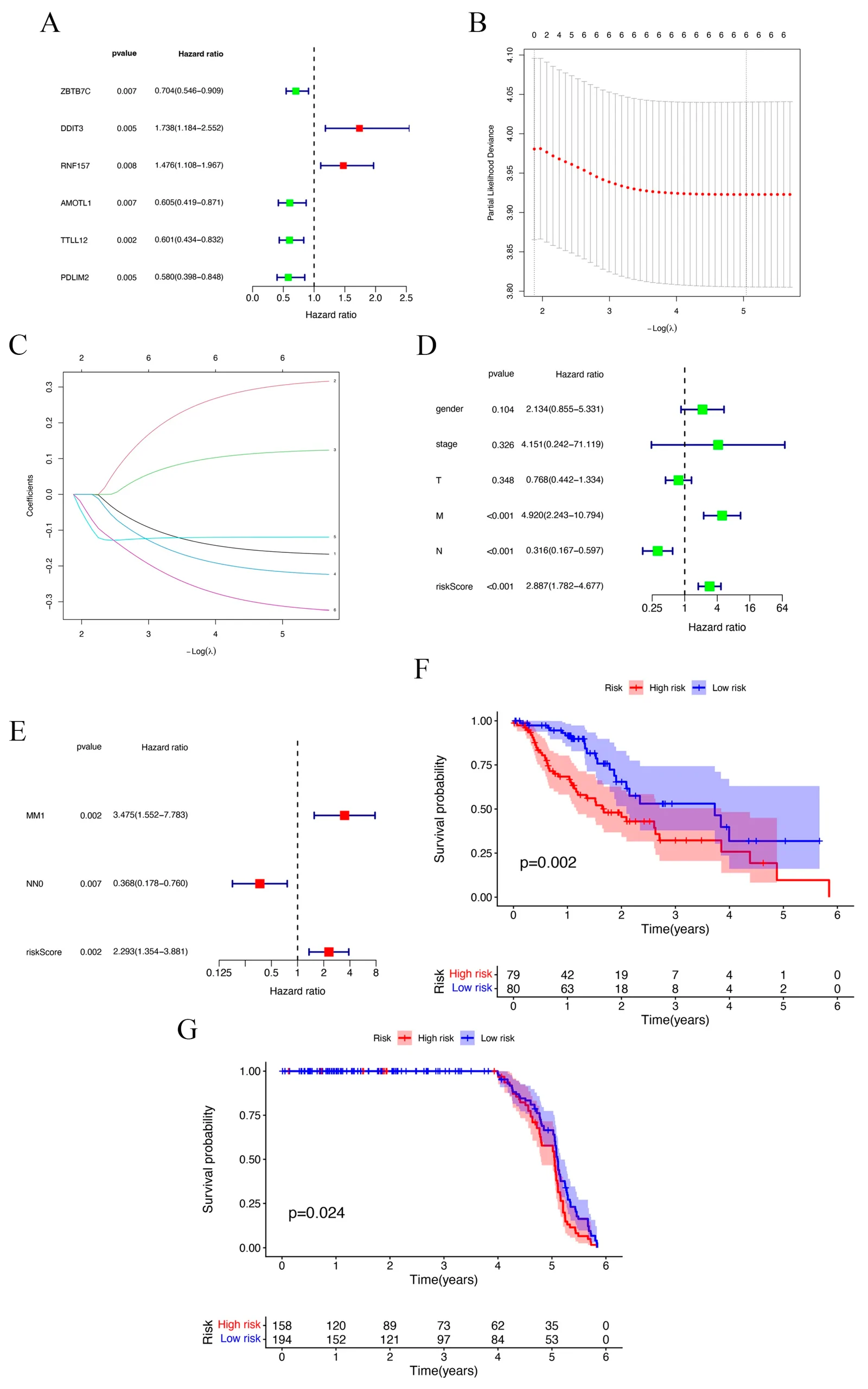
Construction and survival evaluation of the six-gene muscle wasting-related prognostic signature. (**A**) Forest plot of univariate Cox regression analysis identifying six prognostic genes: ZBTB7C, DDIT3, RNF157, AMOTL1, TTLL12, and PDLIM2. Hazard ratios and 95% confidence intervals are shown. (**B**) Selection of the optimal penalty parameter by LASSO-Cox regression. (**C**) LASSO coefficient profiles of the six prognostic genes. (**D**) Multivariate Cox regression analysis of the risk score and clinicopathological variables in the training cohort. (**E**) Cox regression analysis of the risk score in the validation cohort. (**F**) Kaplan–Meier overall survival curves for the high- and low-risk groups in the training cohort. (**G**) Kaplan–Meier overall survival curves for the high- and low-risk groups in the validation cohort.

Univariate Cox regression identified six genes significantly associated with overall survival: ZBTB7C, DDIT3, RNF157, AMOTL1, TTLL12, and PDLIM2 (Figure 2A). ZBTB7C (HR = 0.704, 95% CI: 0.546–0.909), AMOTL1 (HR = 0.605, 95% CI: 0.419–0.871), TTLL12 (HR = 0.601, 95% CI: 0.434–0.832), and PDLIM2 (HR = 0.580, 95% CI: 0.398–0.846) were associated with reduced mortality risk, whereas DDIT3 (HR = 1.738, 95% CI: 1.184–2.552) and RNF157 (HR = 1.476, 95% CI: 1.108–1.967) were associated with increased mortality risk.

LASSO-Cox regression was applied to minimize overfitting and retained all six genes in the final prognostic model (Figure 2B,C). Patients were subsequently stratified into high- and low-risk groups according to the calculated risk score. In multivariable Cox analysis, the risk score remained significantly associated with overall survival after adjustment for available clinicopathological variables (HR = 2.887, 95% CI: 1.782–4.677, *p* < 0.001; Figure 2D). A similar association was observed in the external validation cohort (HR = 2.293, 95% CI: 1.354–3.881, *p* = 0.002; Figure 2E). Kaplan–Meier analysis demonstrated significantly poorer survival in the high-risk group in the training cohort (*p* = 0.002; Figure 2F). The association was also retained in the external cohort, in which patients classified as high risk had significantly poorer survival than those classified as low risk (*p* = 0.024; Figure 2G). Importantly, the six-gene signature was constructed using overall survival as the clinical endpoint, and the candidate genes were derived from a literature-curated muscle wasting-related gene set rather than directly measured skeletal muscle phenotypes. Therefore, these prognostic associations do not establish a direct relationship between the risk score and clinically documented skeletal muscle depletion or cachexia. Furthermore, because the signature was initially developed using the complete TCGA-ESCA cohort, which included both ESCC and EAC, its prognostic associations cannot be assumed to be equivalent across histological subtypes. Although the signature was subsequently evaluated in the independent ESCC cohort GSE53625, further histology-specific validation is required.

### 3.3. Evaluation of the Prognostic Performance of the Six-Gene Signature

**Figure 3:**
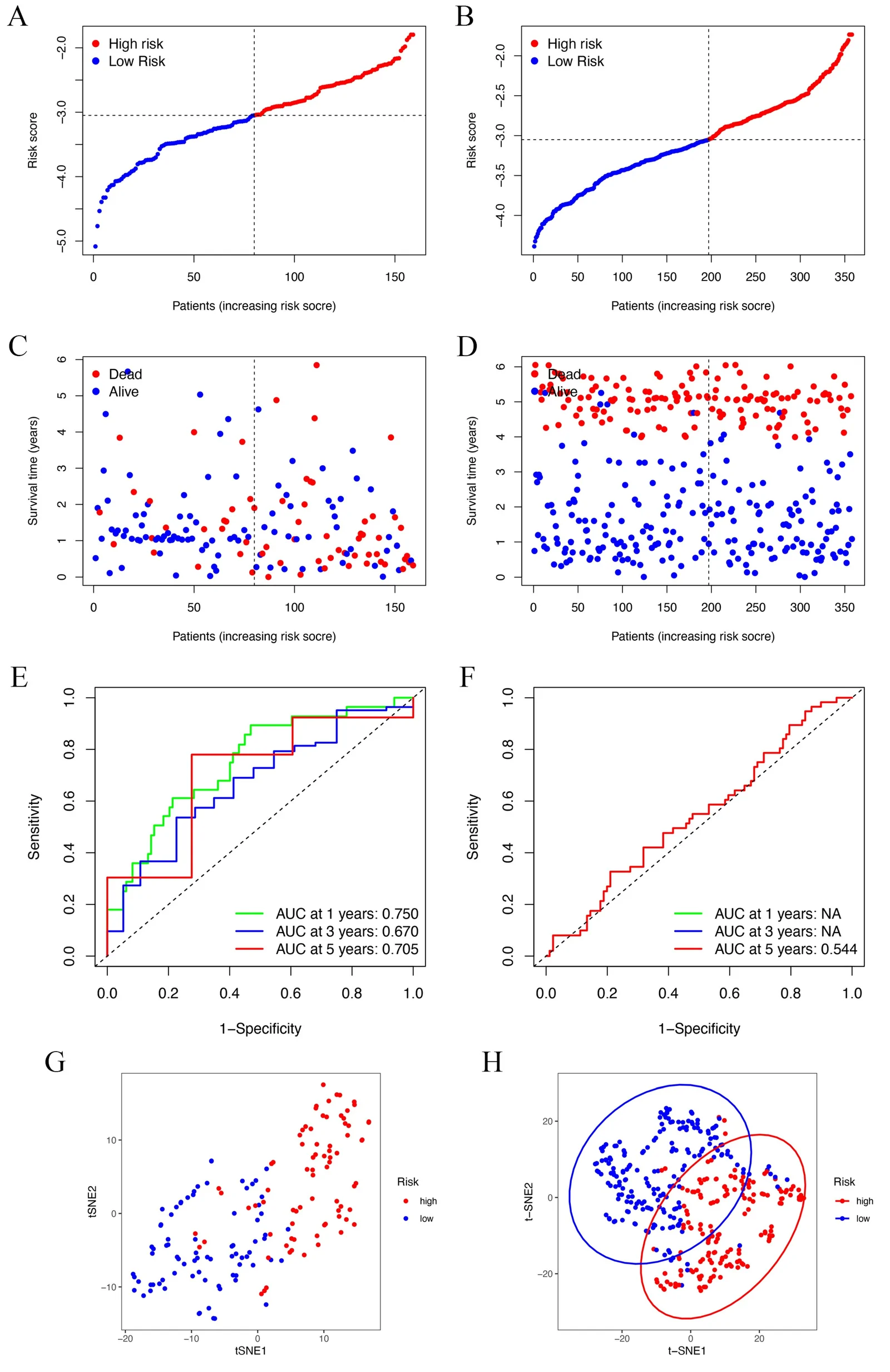
Prognostic performance of the six-gene risk signature in the training and validation cohorts. (**A**,**B**) Distribution of risk scores in the training cohort (**A**) and validation cohort (**B**), with patients ranked according to increasing risk score. (**C**,**D**) Distribution of survival status and survival time according to risk score in the training cohort (**C**) and validation cohort (**D**). (**E**) Time-dependent ROC curves at 1, 3, and 5 years in the training cohort, with AUC values of 0.750, 0.670, and 0.705, respectively. (**F**) Time-dependent ROC analysis in the GSE53625 validation cohort. The available 5-year AUC was 0.544, indicating limited discriminatory performance. The 1- and 3-year AUC values were not available from the present analysis. (**G**,**H**) t-SNE visualization of high- and low-risk patients in the training cohort (**G**) and validation cohort (**H**).

Risk-score distributions and survival status plots showed that deaths became more frequent with increasing risk score in the TCGA training cohort (Figure 3A,C). A similar overall pattern was observed in the GSE53625 validation cohort (Figure 3B,D).

Time-dependent ROC analysis in the training cohort yielded AUC values of 0.750, 0.670, and 0.705 at 1, 3, and 5 years, respectively, indicating moderate prognostic discrimination (Figure 3E). However, the 5-year AUC in the external validation cohort was only 0.544 (Figure 3F), indicating substantially weaker discriminatory performance in the independent ESCC dataset. Although statistically significant survival differences were observed between the high- and low-risk groups, these findings should not be interpreted as evidence of strong individual-level prognostic prediction. Dimensionality reduction analysis further showed separation between the risk groups in the training cohort, whereas separation was less pronounced in the validation cohort (Figure 3G,H).

Taken together, these findings indicate that the six-gene signature was associated with overall survival but exhibited limited external discriminatory performance. Moreover, because risk groups were defined using cohort-specific median cutoffs, the present external evaluation does not establish the transportability of a fixed risk classification threshold. Additional validation is required before the signature can be considered suitable for clinical prognostic stratification.

### 3.4. Biological and Immune Characteristics Associated with the Prognostic Risk Groups

**Figure 4:**
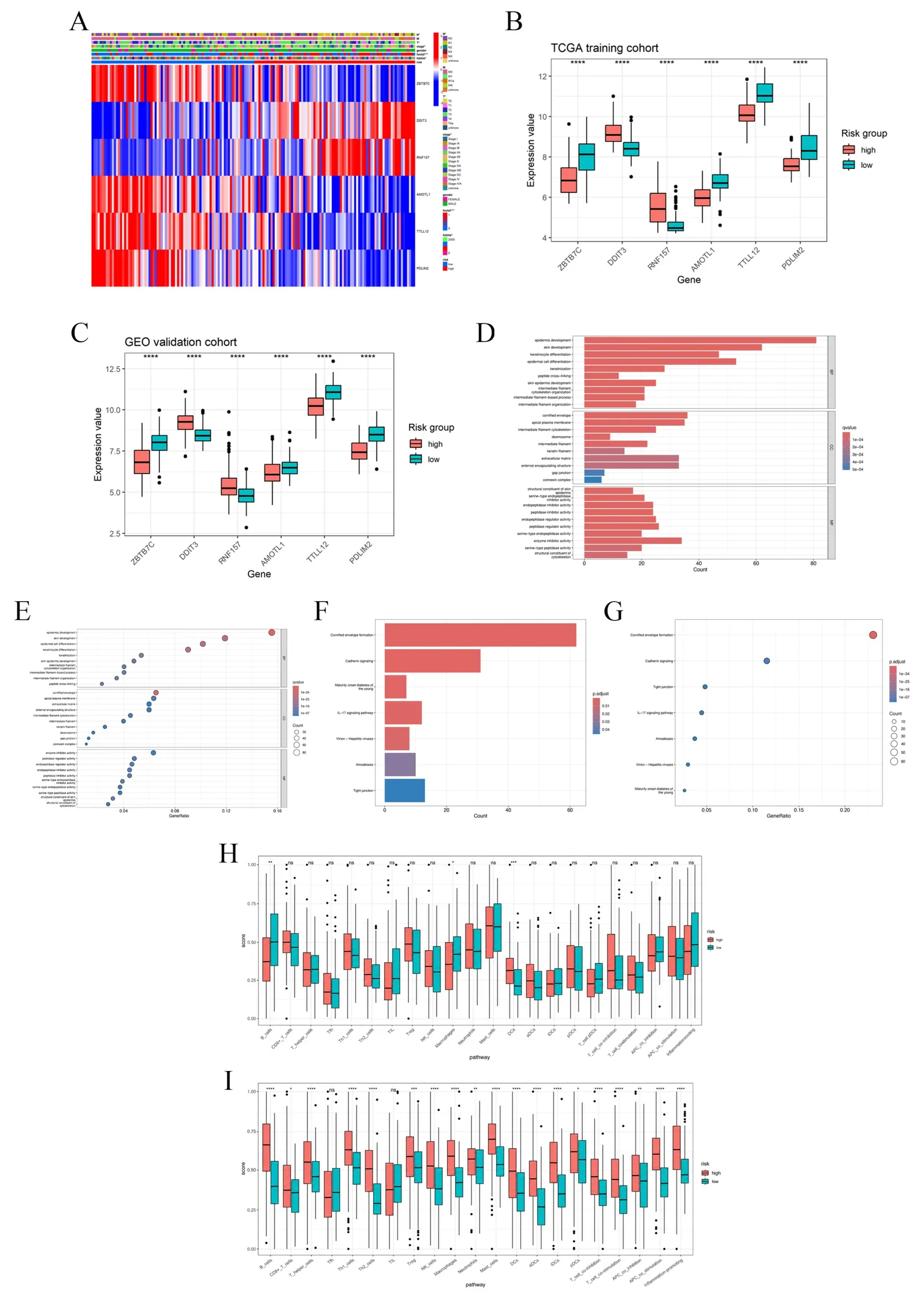
Molecular, functional, and immune characteristics of the high- and low-risk groups. (**A**) Heatmap showing the expression patterns of the six prognostic genes and associated clinical characteristics across patients. (**B**) Comparison of ZBTB7C, DDIT3, RNF157, AMOTL1, TTLL12, and PDLIM2 expression between the high- and low-risk groups in the TCGA training cohort. (**C**) Expression of the six prognostic genes in the high- and low-risk groups in the GEO validation cohort. (**D**,**E**) Gene Ontology enrichment analysis of differentially expressed genes between the high- and low-risk groups, displayed as a bar plot (**D**) and bubble plot (**E**) for biological process, cellular component, and molecular function categories. (**F**,**G**) KEGG pathway enrichment analysis displayed as a bar plot (**F**) and bubble plot (**G**). (**H**,**I**) Comparison of ssGSEA immune-related scores between the high- and low-risk groups in the training and validation cohorts, respectively. ns, not significant; * *p* < 0.05, ** *p* < 0.01, *** *p* < 0.001, **** *p* < 0.0001.

The six prognostic genes exhibited distinct expression patterns between the high- and low-risk groups (Figure 4A). Consistent with their Cox regression coefficients, DDIT3 and RNF157 were expressed at higher levels in the high-risk group, whereas ZBTB7C, AMOTL1, TTLL12, and PDLIM2 were relatively enriched in the low-risk group. These expression patterns were reproducible in both the TCGA training cohort and the GEO validation cohort (Figure 4B,C).

Functional enrichment analysis of genes differentially expressed between the risk groups demonstrated prominent enrichment of biological processes related to epidermis development, keratinocyte differentiation, keratinization, intermediate filament organization, and epithelial structural organization (Figure 4D,E). Cellular-component terms included the cornified envelope, intermediate and keratin filaments, desmosomes, extracellular matrix, and cell junction-related structures, whereas molecular-function terms were enriched for structural constituents and protease/peptidase inhibitor activities. Pathway analyses further highlighted processes involving cornified envelope formation, cadherin signaling, tight junctions, and IL-17-related signaling (Figure 4F,G).

ssGSEA analysis demonstrated broad differences in immune-cell infiltration and immune-related functional scores between the two risk groups (Figure 4H,I), suggesting that the muscle wasting-related prognostic signature was also associated with variation in the tumor immune microenvironment.

### 3.5. ZBTB7C Is Downregulated in Esophageal Cancer and Is Associated with Favorable Prognosis

**Figure 5:**
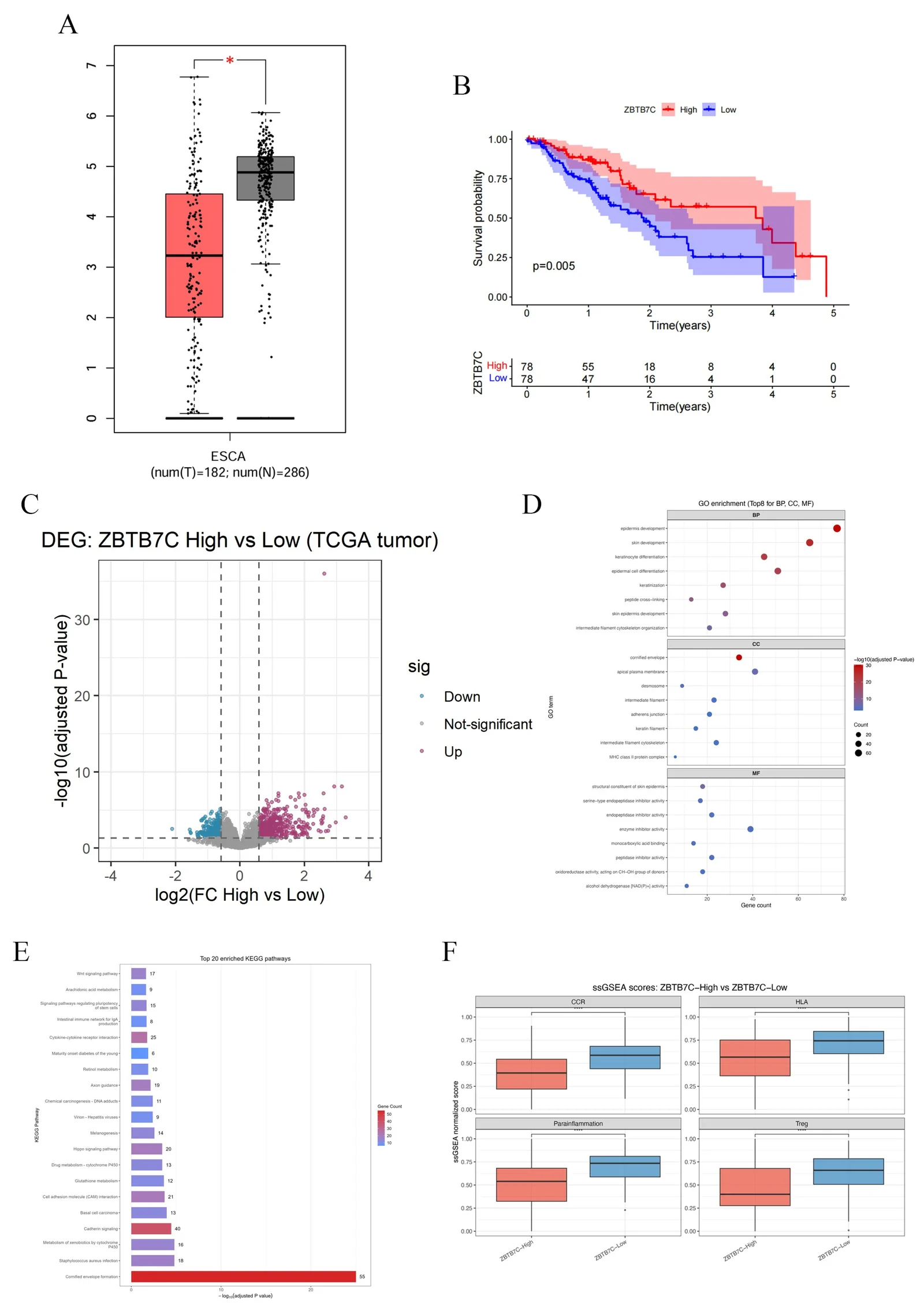
Expression, prognostic significance, and functional characteristics associated with ZBTB7C in esophageal cancer. (**A**) Comparison of ZBTB7C expression between esophageal cancer tumor and normal samples. The number of tumor and normal samples is indicated in the figure. (**B**) Kaplan–Meier overall survival analysis according to high and low ZBTB7C expression. (**C**) Volcano plot of differentially expressed genes between ZBTB7C-high and ZBTB7C-low tumor samples. (**D**) GO enrichment analysis of differentially expressed genes showing the top enriched terms in BP, CC, and MF categories. (**E**) Top 20 enriched KEGG pathways associated with differential ZBTB7C expression. (**F**) Comparison of CCR, HLA, parainflammation, and Treg ssGSEA scores between the ZBTB7C-high and ZBTB7C-low groups. * *p* < 0.05 and **** *p* < 0.0001.

Among the six genes included in the prognostic signature, ZBTB7C was selected for further exploratory investigation based on its significant protective association with overall survival, differential expression in esophageal cancer, known transcriptional regulatory function, and the limited understanding of its potential involvement in tumor-associated skeletal muscle alterations. Notably, ZBTB7C was not the gene with the strongest protective association, as AMOTL1, TTLL12, and PDLIM2 also exhibited hazard ratios below 1 (Figure 2A). Therefore, its selection reflected a combination of prognostic and biological considerations rather than a ranking based solely on effect size. Analysis of esophageal cancer transcriptomic data demonstrated significantly lower ZBTB7C expression in tumor samples than in non-tumor samples (Figure 5A). Kaplan–Meier analysis further showed that patients with higher ZBTB7C expression had significantly better overall survival than those with lower expression (*p* = 0.005; Figure 5B).

Comparison of ZBTB7C-high and ZBTB7C-low tumors identified a substantial set of differentially expressed genes (Figure 5C). GO enrichment analysis predominantly implicated epidermal differentiation, keratinization, intermediate filament organization, cell-junction structures, and protease inhibitor-related molecular functions (Figure 5D). Pathway enrichment analysis also identified multiple epithelial, metabolic, inflammatory, and cell-interaction-related pathways (Figure 5E).

Immune-related analysis revealed significantly higher CCR, HLA, parainflammation, and Treg ssGSEA scores in the ZBTB7C-low group than in the ZBTB7C-high group (Figure 5F), indicating that differences in ZBTB7C expression were accompanied by distinct immune-related characteristics.

### 3.6. Experimental Validation of Reduced ZBTB7C Expression in ESCC

**Figure 6:**
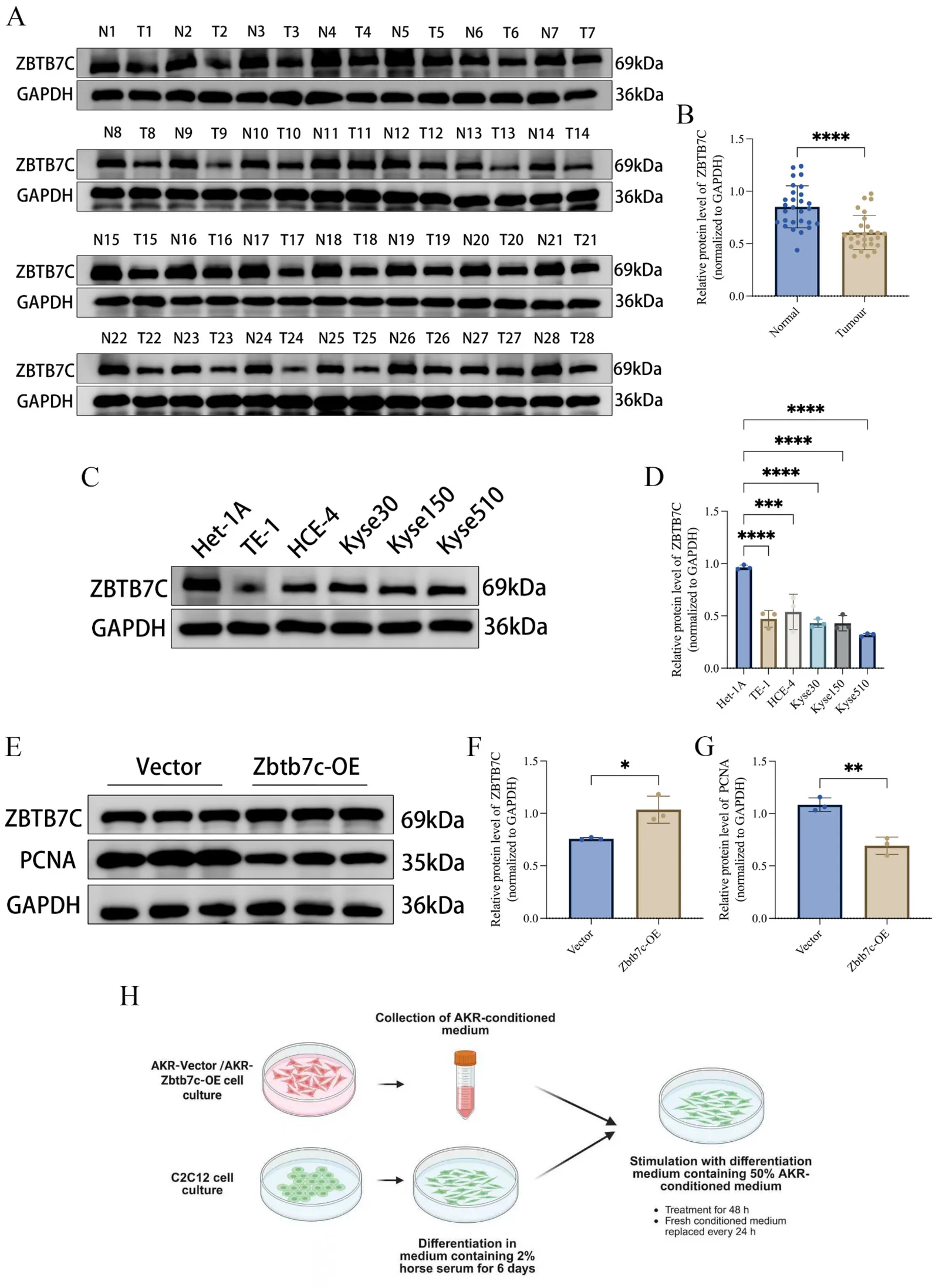
Experimental validation of ZBTB7C expression and establishment of the conditioned-medium model. (**A**) Representative Western blot analysis of ZBTB7C protein expression in paired ESCC tumor tissues (The untrimmed original Western blotting images see Supplementary File S1) (T) and adjacent non-tumor tissues (N) obtained from 28 patients. GAPDH was used as the loading control. (**B**) Densitometric quantification of ZBTB7C protein expression normalized to GAPDH in 28 matched tumor–non-tumor tissue pairs. Each patient contributed one independent paired biological sample. (**C**) Representative Western blot analysis of ZBTB7C expression in the immortalized human esophageal epithelial cell line Het-1A and the ESCC cell lines TE-1, HCE-4, KYSE30, KYSE150, and KYSE510. (**D**) Quantification of ZBTB7C protein expression in the indicated cell lines. (**E**) Representative Western blot analysis of ZBTB7C and PCNA expression in AKR-Vector and AKR-Zbtb7c-OE cells, with GAPDH used as the loading control. (**F**,**G**) Densitometric quantification of ZBTB7C (**F**) and PCNA (**G**) protein expression normalized to GAPDH in AKR-Vector and AKR-Zbtb7c-OE cells. (**H**) Schematic illustration of the AKR-conditioned medium treatment of C2C12 myotubes. C2C12 cells were differentiated for 6 days and subsequently treated for 48 h with Control medium, AKR-Vector-conditioned medium (Vector-CM), or AKR-Zbtb7c-OE-conditioned medium (Zbtb7c-OE-CM), with fresh treatment medium replaced every 24 h. Data are presented as mean ± SD. * *p* < 0.05, ** *p* < 0.01, *** *p* < 0.001 **** *p* < 0.0001, as indicated.

To experimentally validate the expression findings from the bioinformatic analyses, ZBTB7C protein expression was examined in 28 paired ESCC tumor tissues and corresponding adjacent non-tumor tissues obtained from 28 patients. Western blot analysis demonstrated significantly lower normalized ZBTB7C protein expression in tumor tissues than in their matched non-tumor counterparts (*p* < 0.0001; Figure 6A,B). These findings provide independent protein-level evidence of reduced ZBTB7C expression in ESCC tissues. However, the analysis was intended for paired expression validation and does not establish associations between ZBTB7C expression and individual clinicopathological characteristics or skeletal muscle wasting phenotypes.

ZBTB7C expression was subsequently evaluated in the immortalized esophageal epithelial cell line Het-1A and five ESCC cell lines, including TE-1, HCE-4, KYSE30, KYSE150, and KYSE510. ZBTB7C protein expression was consistently lower in the ESCC cell lines than in Het-1A cells, although the magnitude of reduction varied among cell lines (Figure 6C,D).

To establish a model for subsequent functional experiments, Zbtb7c was overexpressed in murine AKR esophageal cancer cells. Western blot analysis confirmed a statistically significant but relatively modest increase in ZBTB7C protein expression (approximately 1.3-fold) in AKR-Zbtb7c-OE cells compared with Vector control cells (*p* < 0.05; Figure 6E,F). This increase was accompanied by a significant reduction in the proliferation-associated protein PCNA (*p* < 0.01; Figure 6E,G). These findings indicate that moderate Zbtb7c overexpression was associated with reduced PCNA expression in AKR cells, although a direct regulatory relationship between ZBTB7C and PCNA was not established. Conditioned media from AKR-Vector and AKR-Zbtb7c-OE cells were subsequently collected and applied to differentiated C2C12 myotubes to evaluate tumor cell-induced muscle wasting-associated responses (Figure 6H).

### 3.7. Zbtb7c Overexpression Attenuates AKR-Conditioned Medium-Induced Alterations in Muscle Protein Homeostasis in C2C12 Myotubes

**Figure 7:**
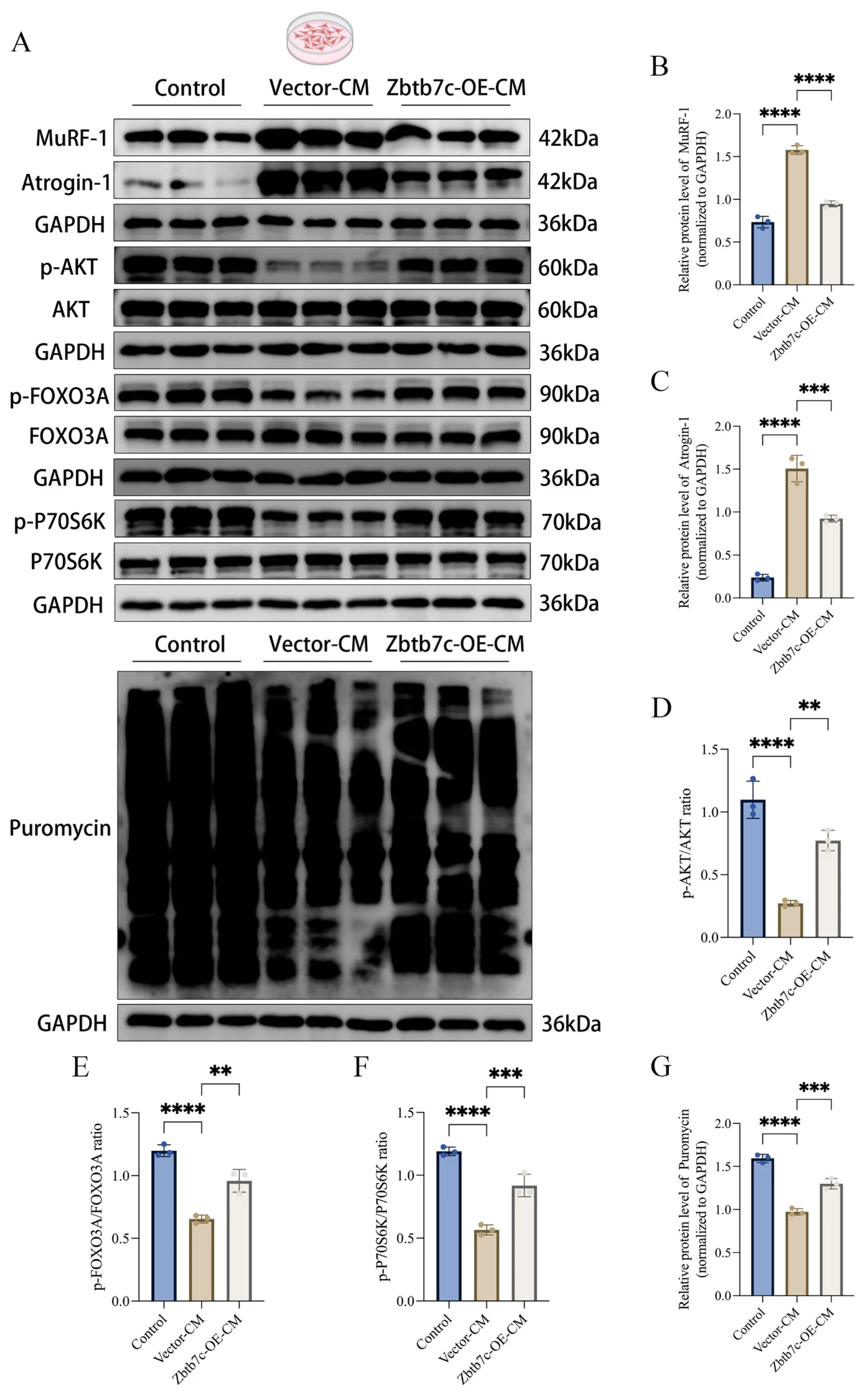
Zbtb7c overexpression attenuates AKR-conditioned medium-induced alterations in muscle protein homeostasis-related markers in C2C12 myotubes. (**A**) Western blot analysis of MuRF-1, Atrogin-1, p-AKT, AKT, p-FOXO3A, FOXO3A, p-P70S6K, P70S6K, and puromycin incorporation in Control, Vector-CM, and Zbtb7c-OE-CM-treated C2C12 myotubes. GAPDH was used as the loading control. (**B**,**C**) Quantification of MuRF-1 (**B**) and Atrogin-1 (**C**) protein expression. (**D**–**F**) Densitometric quantification of protein phosphorylation expressed as the ratios of phosphorylated to corresponding total protein: p-AKT/AKT (**D**), p-FOXO3A/FOXO3A (**E**), and p-P70S6K/P70S6K (**F**). (**G**) Relative global protein synthesis assessed by SUnSET analysis, with the integrated intensity of puromycin-positive bands normalized to GAPDH. ** *p* < 0.01, *** *p* < 0.001, **** *p* < 0.0001, as indicated.

Exposure of differentiated C2C12 myotubes to AKR-Vector-conditioned medium markedly increased the expression of the muscle atrophy-associated ubiquitin ligases MuRF-1 and Atrogin-1 compared with untreated controls (both *p* < 0.0001; Figure 7A–C). In contrast, conditioned medium derived from Zbtb7c-overexpressing AKR cells significantly reduced MuRF-1 and Atrogin-1 expression compared with Vector-CM, although their levels were not completely restored to those of untreated controls.

Vector-CM also markedly suppressed AKT phosphorylation, as reflected by a decreased p-AKT/AKT ratio. This reduction was partially reversed by Zbtb7c-OE-CM (*p* < 0.01 versus Vector-CM; Figure 7D). A similar pattern was observed for FOXO3A phosphorylation at Ser253. Vector-CM decreased the p-FOXO3A (Ser253)/total FOXO3A ratio, whereas Zbtb7c-OE-CM significantly increased phosphorylation at this site compared with Vector-CM (Figure 7E). These changes occurred alongside corresponding differences in MuRF-1 and Atrogin-1 expression. In parallel, the p-P70S6K/P70S6K ratio was reduced by Vector-CM and partially recovered following exposure to Zbtb7c-OE-CM (Figure 7F).

SUnSET analysis using puromycin incorporation showed a pronounced reduction in global protein synthesis after Vector-CM treatment. Zbtb7c-OE-CM significantly increased puromycin incorporation compared with Vector-CM (*p* < 0.001; Figure 7G). Collectively, these findings indicate that Zbtb7c overexpression in AKR tumor cells attenuated conditioned medium-induced activation of muscle proteolytic markers and partially restored protein synthesis-related signaling in C2C12 myotubes. These molecular findings suggest changes in muscle protein homeostasis but do not directly establish morphological myotube atrophy or its attenuation. Because conditioned media were standardized by their proportion in the final treatment medium rather than by the number of viable tumor cells contributing to their production, these findings cannot distinguish differences in specific tumor-derived factors from potential effects of altered tumor-cell proliferation or viability.

### 3.8. Zbtb7c Overexpression Suppresses Tumor Growth and Is Associated with Preservation of Skeletal Muscle Mass and Function In Vivo

**Figure 8:**
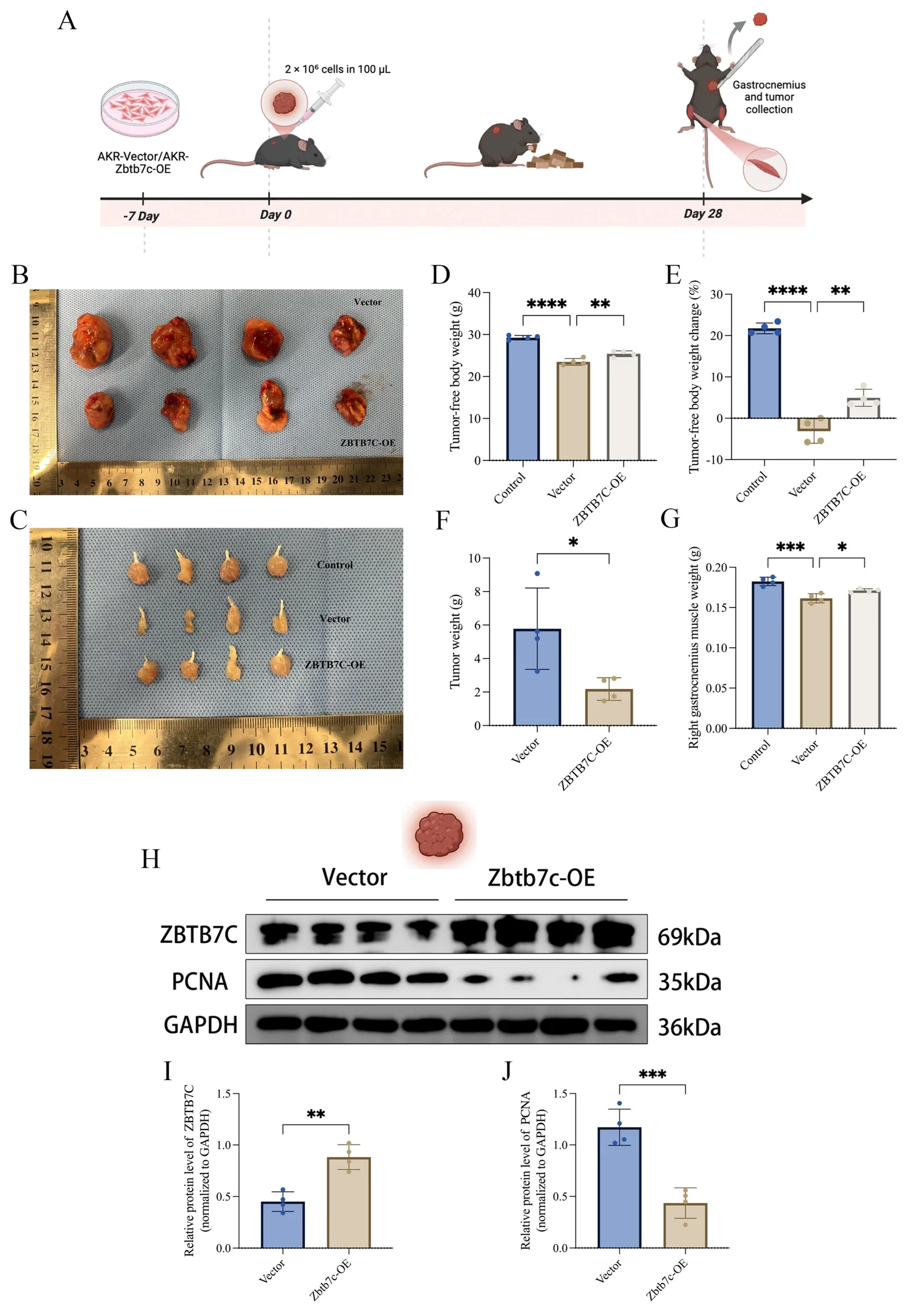
Zbtb7c overexpression suppresses tumor growth and preserves gastrocnemius muscle mass in vivo. (**A**) Schematic of the animal experimental protocol. After a 7-day acclimatization period, mice received AKR-Vector or AKR-Zbtb7c-OE cells (2 × 10^6^ cells in 100 μL) on day 0, and tumors and gastrocnemius muscles were collected on day 28. (**B**) Representative images of tumors from the Vector and Zbtb7c-OE groups. (**C**) Representative images of gastrocnemius muscles from the Control, Vector, and Zbtb7c-OE groups. (**D**) Tumor-free body weight at the experimental endpoint. (**E**) Percentage change in tumor-free body weight. (**F**) Tumor weight in the Vector and Zbtb7c-OE groups. (**G**) Right gastrocnemius muscle weight in the Control, Vector, and Zbtb7c-OE groups. (**H**) Western blot analysis of ZBTB7C and PCNA in tumor tissues from the Vector and Zbtb7c-OE groups. GAPDH was used as the loading control. (**I**,**J**) Quantification of ZBTB7C (**I**) and PCNA (**J**) protein expression in tumor tissues. * *p* < 0.05, ** *p* < 0.01, *** *p* < 0.001, **** *p* < 0.0001.

The effects of tumor-cell Zbtb7c overexpression were further evaluated using an immunocompetent AKR/C57BL/6 syngeneic tumor model, with tissues collected at the experimental endpoint on day 28 (Figure 8A). Macroscopic examination showed smaller tumors in mice bearing Zbtb7c-OE tumors than in the Vector group (Figure 8B). In parallel, gastrocnemius muscles from tumor-bearing mice appeared reduced compared with those from non-tumor controls, whereas this reduction was partially attenuated in the Zbtb7c-OE group (Figure 8C).

Tumor-bearing Vector mice exhibited a marked reduction in tumor-free body weight compared with non-tumor controls (*p* < 0.0001). Zbtb7c overexpression significantly attenuated this reduction (*p* < 0.01 versus Vector; Figure 8D). A similar pattern was observed for the percentage change in tumor-free body weight, with the Vector group showing weight loss whereas the Zbtb7c-OE group showed partial preservation of body weight (*p* < 0.01; Figure 8E).

Tumor weight was significantly lower in the Zbtb7c-OE group than in the Vector group (*p* < 0.05; Figure 8F). Importantly, right gastrocnemius muscle weight was reduced in Vector tumor-bearing mice relative to controls (*p* < 0.001) and was significantly higher in the Zbtb7c-OE group than in the Vector group (*p* < 0.05; Figure 8G). To further evaluate skeletal muscle function, longitudinal all-limb grip strength was assessed on days 0, 7, 14, 21, and 28 (Supplementary Figure S1A). Grip strength progressively increased in non-tumor-bearing Control mice but declined in Vector tumor-bearing mice. In contrast, mice bearing Zbtb7c-overexpressing tumors exhibited a smaller decline in grip strength over the experimental period. Analysis of the percentage change from baseline to day 28 demonstrated a significant reduction in the Vector group compared with the Control group (*p* < 0.0001). Importantly, the reduction in grip strength was significantly attenuated in the Zbtb7c-OE group compared with the Vector group (*p* < 0.01; Supplementary Figure S1B). These findings indicate that Zbtb7c overexpression was associated with partial preservation of skeletal muscle function, accompanied by reduced tumor burden and preservation of gastrocnemius muscle mass.

Western blot analysis of tumor tissues confirmed sustained ZBTB7C overexpression in vivo (*p* < 0.01) and demonstrated a marked reduction in PCNA expression (*p* < 0.001) in Zbtb7c-OE tumors compared with Vector tumors (Figure 8H–J). Collectively, these findings indicate that Zbtb7c overexpression in AKR tumor cells was associated with reduced tumor burden, preservation of tumor-free body weight and gastrocnemius muscle mass, and attenuation of the decline in all-limb grip strength. However, because tumor growth suppression and muscle-related improvements occurred concurrently, the present results do not establish whether these effects were independent of differences in tumor burden.

### 3.9. Zbtb7c Overexpression Ameliorates Muscle Wasting-Associated Molecular Alterations In Vivo

**Figure 9:**
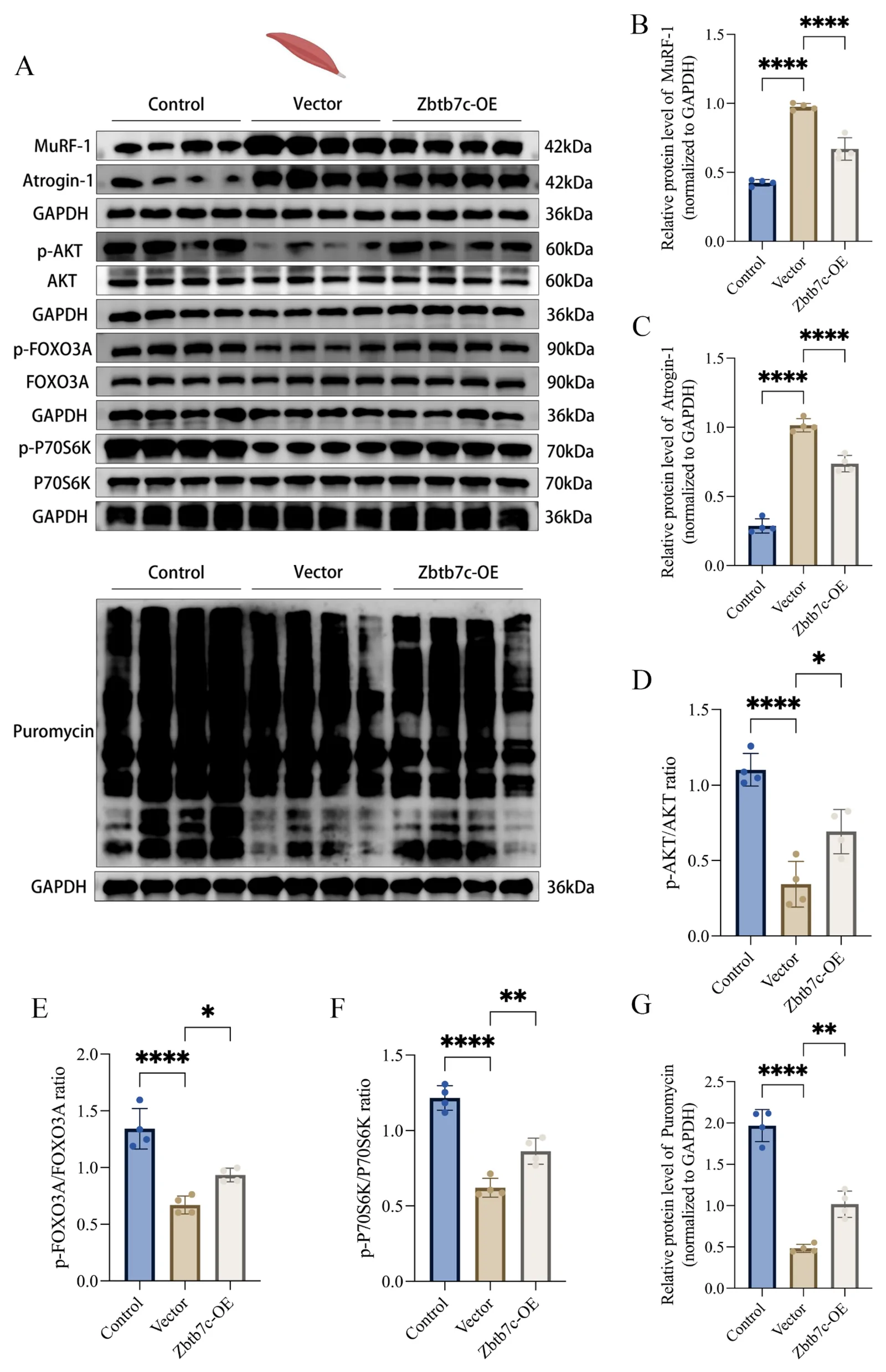
Zbtb7c overexpression attenuates skeletal muscle wasting-associated molecular alterations in tumor-bearing mice. (**A**) Western blot analysis of MuRF-1, Atrogin-1, p-AKT, AKT, p-FOXO3A, FOXO3A, p-P70S6K, P70S6K, and puromycin incorporation in gastrocnemius muscle from the Control, Vector, and Zbtb7c-OE groups. GAPDH was used as the loading control. (**B**,**C**) Quantification of MuRF-1 (**B**) and Atrogin-1 (**C**) protein expression. (**D**–**F**) Densitometric quantification of phosphorylation expressed as p-AKT/AKT (**D**), p-FOXO3A/FOXO3A (**E**), and p-P70S6K/P70S6K (**F**) ratios. (**G**) Relative global protein synthesis assessed by SUnSET analysis, with the integrated intensity of puromycin-positive bands normalized to GAPDH. * *p* < 0.05, ** *p* < 0.01, **** *p* < 0.0001.

Consistent with the reduction in gastrocnemius muscle mass, skeletal muscle from Vector tumor-bearing mice showed marked activation of muscle catabolic signaling (Figure 9A). MuRF-1 and Atrogin-1 protein levels were substantially increased compared with non-tumor controls (both *p* < 0.0001). These increases were significantly attenuated in mice bearing Zbtb7c-OE tumors (Figure 9B,C).

Tumor burden was also associated with pronounced suppression of AKT signaling, with the p-AKT/AKT ratio markedly reduced in the Vector group. Zbtb7c overexpression partially restored AKT phosphorylation (*p* < 0.05 versus Vector; Figure 9D). Similarly, the p-FOXO3A (Ser253)/total FOXO3A ratio was reduced in Vector tumor-bearing mice and was significantly higher in the Zbtb7c-OE group (*p* < 0.05; Figure 9E). This change was accompanied by lower MuRF-1 and Atrogin-1 expression in the Zbtb7c-OE group. The p-P70S6K/P70S6K ratio followed the same pattern, with significant recovery after Zbtb7c overexpression (*p* < 0.01; Figure 9F).

SUnSET analysis further demonstrated a substantial reduction in skeletal muscle protein synthesis in Vector tumor-bearing mice compared with controls. Puromycin incorporation was significantly increased in the Zbtb7c-OE group compared with the Vector group (*p* < 0.01; Figure 9G). Together, the in vivo findings were consistent with the C2C12 conditioned-medium experiments and showed that tumor-cell Zbtb7c overexpression was accompanied by reduced muscle proteolytic signaling and partial restoration of anabolic/protein synthesis-associated signaling.

## 4. Discussion

In the present study, we integrated transcriptomic analysis with experimental validation to investigate muscle wasting-related molecular features in esophageal cancer. Using the complete TCGA-ESCA cohort [20], we identified 32 muscle wasting-related genes and established a six-gene prognostic signature comprising ZBTB7C, DDIT3, RNF157, AMOTL1, TTLL12, and PDLIM2. Among these candidates, ZBTB7C was prioritized for exploratory experimental investigation based on its prognostic association, differential expression, known transcriptional regulatory function, and the limited understanding of its potential involvement in tumor-associated skeletal muscle alterations. This selection was biologically informed rather than based on a formal comparative ranking, and does not imply that ZBTB7C was the strongest prognostic or most biologically influential gene within the signature. Experimental validation further demonstrated reduced ZBTB7C expression in human ESCC tissues and cell lines, while Zbtb7c overexpression in AKR tumor cells was associated with reduced PCNA expression and attenuation of muscle wasting-associated changes in both cellular and animal models.

An important distinction in interpreting the present findings is that the six-gene prognostic signature was derived from a literature-curated muscle wasting-related gene set rather than from patients with directly documented skeletal muscle depletion or cachexia. Although these genes were selected based on their previously reported involvement in muscle wasting-related biological processes, the TCGA-ESCA and GSE53625 cohorts did not contain corresponding individual-level measurements of skeletal muscle mass, longitudinal body-composition changes, or cachexia status. Consequently, the observed survival associations cannot establish that the signature specifically reflects skeletal muscle wasting rather than broader aspects of tumor biology and prognosis. This distinction is particularly relevant because clinically meaningful muscle wasting in ESCC is generally evaluated using objective body-composition measurements and functional or nutritional assessments. For example, Zhao et al. [15]. reported that longitudinal changes in CT-derived skeletal muscle index were associated with pathological response to neoadjuvant immunotherapy plus chemotherapy and postoperative complications in patients with ESCC. These observations emphasize the importance of integrating molecular findings with directly measured skeletal muscle phenotypes. Future studies incorporating CT-derived skeletal muscle index, longitudinal muscle loss, nutritional status, and functional outcomes will be necessary to determine whether the six-gene signature or ZBTB7C provides information specifically related to muscle wasting beyond conventional prognostic factors.

The six-gene signature was associated with overall survival in both the TCGA training cohort and the independent GSE53625 validation cohort. However, its prognostic discrimination was substantially weaker in the external cohort, with a 5-year AUC of 0.544 compared with 0.705 in the training cohort. This discrepancy indicates limited external predictive performance despite the statistically significant survival differences between the risk groups. Furthermore, because gene expression values were standardized separately within each cohort and patients were stratified using cohort-specific median risk scores, the external analysis did not fully evaluate the transportability of a prespecified risk classification threshold. Differences in histological composition and gene expression measurement platforms between the training and validation cohorts may also have contributed to the observed variation in model performance, although their individual effects were not formally assessed. Importantly, while multivariable Cox regression indicated that the risk score was associated with survival after adjustment for available clinicopathological variables, this does not establish incremental predictive value over conventional prognostic models. Previous ESCC research has demonstrated that incorporating molecular characteristics into pathological staging may improve prognostic stratification. For example, Jiang et al. reported improved prognostic discrimination when a molecular score was combined with pathological T classification compared with pathological T classification alone [53]. In contrast, the present study did not directly compare the discrimination or calibration of the six-gene signature with conventional clinicopathological models. Therefore, the signature should be regarded as an exploratory prognostic framework that facilitates the identification of biologically relevant candidate genes rather than an optimized clinical prediction tool. Future studies should evaluate fixed training-derived risk thresholds, harmonized expression preprocessing, concordance indices, calibration, and incremental prognostic performance in adequately powered, independent ESCC cohorts.

Among the six genes, ZBTB7C showed particular biological interest. Transcriptomic analysis demonstrated lower ZBTB7C expression in esophageal cancer, and this finding was supported at the protein level in 28 paired ESCC and adjacent non-tumor tissues as well as in multiple ESCC cell lines. Higher ZBTB7C expression was also associated with better overall survival. In AKR cells, Zbtb7c overexpression decreased PCNA expression, while tumors derived from Zbtb7c-overexpressing cells showed reduced tumor weight and lower PCNA expression in vivo. These findings support an association between ZBTB7C and reduced tumor growth [16,54]. Nevertheless, the direct transcriptional targets and tumor-cell mechanisms downstream of ZBTB7C remain to be determined. It should also be emphasized that the assessment of ZBTB7C expression across human ESCC specimens and cell lines does not constitute independent functional validation of its muscle-related effects. Because the functional experiments were primarily performed using the AKR tumor-cell system, the reproducibility of these findings across additional ESCC cell lines and tumor backgrounds remains to be established.

A major finding of this study was that altered Zbtb7c expression in tumor cells was associated with changes in skeletal muscle protein homeostasis-related markers. Conditioned medium from AKR-Vector cells increased MuRF-1 and Atrogin-1 expression in differentiated C2C12 myotubes and decreased the phosphorylation of AKT, FOXO3A, and P70S6K, together with reduced puromycin incorporation. In contrast, conditioned medium from Zbtb7c-overexpressing AKR cells partially attenuated these alterations and increased global protein synthesis. These observations suggest that Zbtb7c expression in tumor cells may influence the effects of tumor-conditioned medium on muscle protein homeostasis-related processes. However, because myotube diameter, area, and myosin heavy-chain staining were not evaluated, the present in vitro experiments do not directly establish morphological myotube atrophy or its attenuation. In the syngeneic mouse model, Zbtb7c overexpression was accompanied by preservation of gastrocnemius muscle mass and changes in muscle catabolic and protein synthesis-related markers. Thus, the animal experiments provide complementary tissue-level evidence, although the observed muscle preservation cannot be fully separated from the reduction in tumor burden. Collectively, these findings support an association between tumor-cell Zbtb7c expression and skeletal muscle protein homeostasis-related alterations rather than establishing a direct protective mechanism against myotube atrophy [7,8,55].

The observed molecular changes are biologically consistent with established regulators of muscle protein homeostasis. AKT–FOXO3A signaling is closely associated with regulation of muscle proteolysis, whereas P70S6K phosphorylation and puromycin incorporation reflect changes related to protein synthesis [55,56,57]. In the present study, the coordinated changes in these molecular markers suggest that differences in tumor-cell Zbtb7c expression may influence muscle catabolic and protein synthesis-related responses to tumor-conditioned medium. However, the specific tumor-derived factors responsible for these effects were not identified, and no neutralization or rescue experiments were performed to establish a causal relationship between candidate mediators and the observed muscle responses. Furthermore, the changes in AKT, FOXO3A, and P70S6K phosphorylation represent downstream molecular associations rather than direct evidence of a defined signaling mechanism. Therefore, the present findings support a potential relationship between tumor-cell Zbtb7c expression and skeletal muscle protein homeostasis but do not establish a specific ZBTB7C-dependent tumor–muscle signaling axis.

The in vivo findings should be interpreted with particular consideration of the concurrent reduction in tumor burden. Zbtb7c overexpression significantly suppressed tumor growth while simultaneously preserving tumor-free body weight and gastrocnemius muscle mass. The additional longitudinal grip strength measurements further demonstrated that mice bearing Zbtb7c-overexpressing tumors exhibited a smaller decline in muscle strength than Vector tumor-bearing mice. These functional findings complement the muscle mass and molecular results, suggesting that the observed skeletal muscle alterations were accompanied by differences in muscle performance. Because reduced tumor burden may alleviate systemic catabolic stress, the observed muscle preservation cannot be conclusively attributed to a specific Zbtb7c-dependent tumor–muscle signaling mechanism. The conditioned-medium experiments provide complementary evidence that differences in tumor-cell Zbtb7c expression are associated with altered effects of tumor-conditioned medium on muscle protein homeostasis-related processes in C2C12 myotubes, without the systemic influence of whole-animal tumor burden. However, these experiments do not establish whether the observed effects result from specific changes in secreted mediators or from differences in tumor-cell proliferation, cell number, or secretory activity during conditioned-medium preparation. Moreover, tumor-burden-matched comparisons were not performed in the present study. Therefore, although the combined cellular and animal findings support an association between tumor-cell Zbtb7c expression and skeletal muscle alterations, they do not establish a tumor-burden-independent muscle-protective mechanism. Further studies using tumor-burden-matched experimental designs and functional validation of candidate tumor-derived mediators will be necessary to distinguish the direct and indirect contributions of Zbtb7c to tumor-associated skeletal muscle changes [58,59,60].

From a clinical perspective, these findings should be interpreted within the broader framework of skeletal muscle assessment and prognostic evaluation in ESCC. Clinically meaningful skeletal muscle wasting is typically characterized using objective measurements of body composition, skeletal muscle index, nutritional status, and physical function. Previous ESCC studies have demonstrated that longitudinal changes in skeletal muscle index are associated with treatment response and postoperative complications [15], while molecular characteristics may provide additional prognostic information when integrated with conventional pathological staging [53]. In the present study, the association of ZBTB7C with survival and tumor-related skeletal muscle alterations provides a rationale for investigating its potential relevance to these clinical outcomes. However, because direct skeletal muscle phenotypes were unavailable in the analyzed patient cohorts and the six-gene signature exhibited limited external discriminatory performance, neither ZBTB7C nor the signature can currently be considered a clinically validated biomarker of muscle wasting or an established improvement over conventional prognostic assessment. Future investigations integrating tumor ZBTB7C expression with CT-derived skeletal muscle measurements, longitudinal body-composition changes, nutritional and functional assessments, and established clinicopathological variables will be necessary to determine its clinical significance.

Several limitations should be acknowledged. First, the bioinformatic discovery and prognostic model-development analyses were based on the complete TCGA-ESCA cohort, which included both ESCC and EAC, whereas subsequent experimental investigations were conducted predominantly in ESCC-related models. Given the substantial biological and clinical differences between these histological subtypes, prognostic associations identified in the mixed TCGA-ESCA cohort cannot be assumed to apply equally to ESCC and EAC. Although the independent GSE53625 cohort provided an external evaluation in ESCC patients, the limited discriminatory performance of the six-gene signature in this cohort underscores the need for further histology-specific validation. Consequently, the experimental implications of the present study should be interpreted primarily in the context of ESCC, while the generalizability of the prognostic findings across different histological subtypes remains uncertain. Second, neither TCGA-ESCA nor GSE53625 contained direct measurements of skeletal muscle mass, sarcopenia, cachexia status, or longitudinal body-composition changes. Therefore, the six-gene signature was developed from literature-curated candidate genes and validated against overall survival, rather than against a directly observed muscle wasting phenotype. Independent validation in clinically characterized cohorts with CT-derived skeletal muscle measurements and cachexia-related variables is required before the signature can be considered a muscle wasting-associated clinical biomarker. Third, although the six-gene signature was externally evaluated, its discriminatory performance was limited in the independent GSE53625 cohort. The use of cohort-specific expression standardization and median risk-score cutoffs limited the assessment of model transportability. Furthermore, concordance indices, calibration, and incremental predictive performance relative to conventional clinicopathological models were not comprehensively evaluated. Consequently, the clinical applicability of the signature remains uncertain and requires further validation using standardized prediction procedures and independent ESCC cohorts. Fourth, the animal experiments included only four mice per group, and no formal prospective sample-size calculation was performed. Given the biological variability in tumor growth, body weight, and skeletal muscle mass, the limited sample size reduces the precision and generalizability of the observed effects. Furthermore, gastrocnemius muscle mass was assessed using absolute tissue weight without normalization to tibia length or an appropriate body-size indicator. Although longitudinal all-limb grip strength measurements provided complementary functional evidence, more comprehensive functional and morphological assessments in larger cohorts would strengthen these findings. In addition, the C2C12 conditioned-medium experiments evaluated muscle atrophy-associated molecular markers and global protein synthesis but did not include direct morphological measurements of myotube diameter, area, or myosin heavy-chain staining. Therefore, the in vitro findings cannot establish that Zbtb7c overexpression prevents or reverses morphological myotube atrophy. Fifth, although ZBTB7C expression was evaluated in human ESCC specimens and multiple ESCC cell lines, the functional experiments investigating tumor-associated skeletal muscle alterations were primarily based on the murine AKR tumor-cell system and differentiated C2C12 myotubes. The muscle-related effects of Zbtb7c overexpression have not been independently validated in additional human ESCC cell lines, human skeletal muscle cell models, or complementary in vivo models. Consequently, the reproducibility of these findings across different tumor backgrounds and their relevance to human ESCC-associated skeletal muscle wasting remain uncertain. Further studies using additional ESCC models and independent genetic perturbation approaches are required before ZBTB7C can be considered a validated therapeutic target for cancer-associated skeletal muscle wasting. Finally, the specific tumor-derived mediators responsible for the observed changes in skeletal muscle protein homeostasis were not identified. Although the conditioned-medium experiments suggest that tumor-cell Zbtb7c expression may influence the effects of soluble tumor-associated factors on skeletal muscle cells, secretome or cytokine profiling and functional validation of candidate mediators were not performed. Furthermore, potential differences in tumor-cell proliferation, cell number, or secretory activity during conditioned-medium preparation may contribute to the observed responses. Together with the concurrent reduction in tumor burden in vivo and the absence of tumor-burden-matched comparisons, these limitations prevent definitive attribution of the muscle-related effects to a specific ZBTB7C-dependent tumor–muscle signaling mechanism. Future studies integrating standardized conditioned-medium preparation, tumor secretome profiling, cytokine analysis, and candidate-factor neutralization or rescue experiments will be necessary to clarify the underlying mechanisms.

Overall, the present study identifies prognostically associated genes derived from a literature-curated muscle wasting-related gene set and provides experimental evidence linking tumor-cell Zbtb7c expression with alterations in skeletal muscle protein homeostasis and muscle-related phenotypes. The cellular experiments demonstrated differences in muscle atrophy-associated molecular markers and puromycin incorporation following exposure to tumor-conditioned medium, while the animal experiments showed concurrent changes in tumor growth, gastrocnemius muscle mass, and all-limb grip strength. Nevertheless, these observations do not establish a specific tumor-derived signaling mechanism, a muscle-protective effect independent of reduced tumor burden, or a clinically validated role for ZBTB7C in predicting or treating cancer cachexia. Further studies incorporating direct clinical muscle phenotyping, additional ESCC models, and mechanistic validation are required to clarify its biological and translational significance.

## 5. Conclusions

In summary, we identified a six-gene prognostic signature derived from a literature-curated muscle wasting-related gene set in esophageal cancer and found that higher ZBTB7C expression was associated with favorable overall survival. However, the signature was not validated against directly measured skeletal muscle wasting phenotypes and exhibited limited external discriminatory performance. In experimental models, Zbtb7c overexpression in AKR tumor cells was associated with reduced tumor growth and attenuated conditioned medium-induced alterations in muscle protein homeostasis-related processes in C2C12 myotubes. In tumor-bearing mice, Zbtb7c overexpression was accompanied by preservation of gastrocnemius muscle mass, a smaller decline in all-limb grip strength, and attenuation of muscle protein homeostasis-related molecular alterations. Nevertheless, the muscle-related effects cannot be fully separated from reduced tumor burden, and the specific tumor-derived mediators and causal signaling mechanisms remain unidentified. These findings support further investigation of ZBTB7C as a candidate associated with tumor-related skeletal muscle alterations in ESCC, rather than establishing its clinical utility as a prognostic biomarker or therapeutic target for cancer-associated muscle wasting.

## Data Availability

The publicly available datasets analyzed in this study were obtained from The Cancer Genome Atlas Esophageal Carcinoma cohort (TCGA-ESCA) and the Gene Expression Omnibus (GEO). The remaining data generated or analyzed during the current study, including Western blotting, cell-based experiments, animal experiments, and other validation data, are available from the corresponding author upon reasonable request.

## Supplementary Materials

The following supporting information can be downloaded at: https://www.mdpi.com/article/10.3390/cancers18213479/s1. Supplementary Figure S1: Zbtb7c overexpression attenuates the decline in all-limb grip strength in tumor-bearing mice; Supplementary Table S1: Muscle wasting-related genes included in the bioinformatic analysis; Supplementary Table S2: Antibodies used for Western blotting. File S1: The untrimmed original Western blotting images.
