# Supplementary material for: A Literature-Derived Muscle Wasting-Related Prognostic Signature Identifies ZBTB7C as a Candidate Associated with Tumor-Related Skeletal Muscle Alterations in Esophageal Cancer
Source: Cancers (Basel). 2026 Oct 28;18(21):3479. doi: 10.3390/cancers18213479 (PMC13762034; doi:10.3390/cancers18213479)
Supplement: Supplementary file 1 [file cancers-18-03479-s001.zip › File S1.pdf]

The original data is arranged based on the sequence of Western Blots data provided in the manuscript.

Marker: Epizyme，WJ103

Figure 6A (N1T1-N7T7)

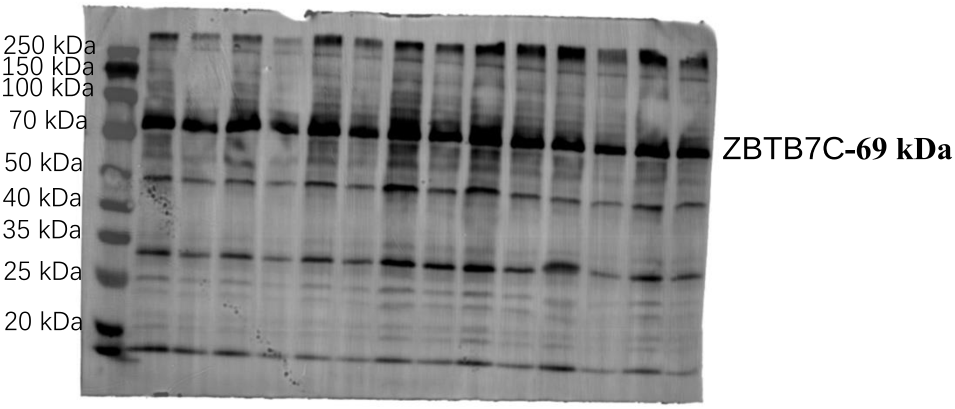

25682.761	22021.69	27567.933	17100.154	31905.983	23181.033	38726.518	29868.861	36385.983	30006.69	24871.518	18191.104	21426.154	17540.397

Figure 6A（N8T8-N14T14）

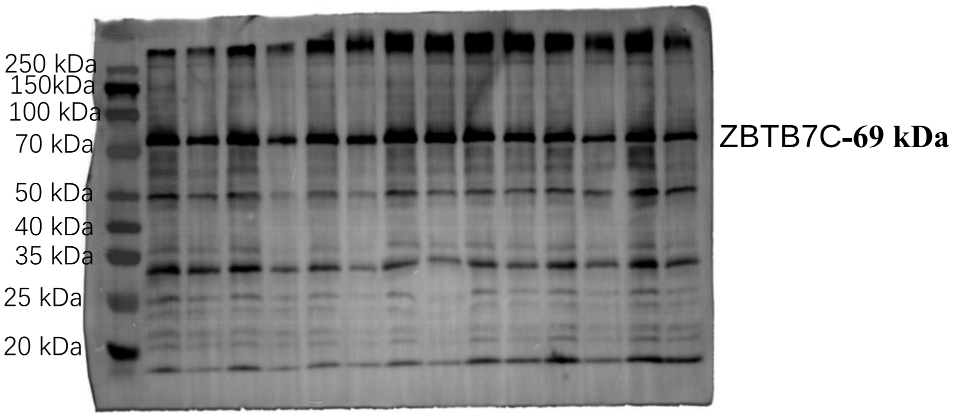

30943.397	20068.154	30115.104	17308.569	28006.569	22111.912	35387.276	33267.983	33398.397	26334.983	27005.154	19270.104	28173.447	22137.418

Figure 6A (N15T15-N21T21)

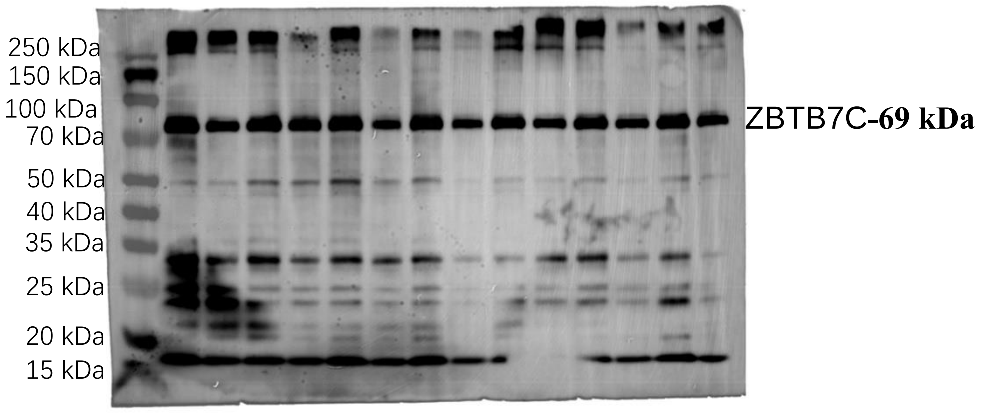

[illegible]	[illegible]	[illegible]	[illegible]	[illegible]	[illegible]	[illegible]	[illegible]	[illegible]	[illegible]	[illegible]	[illegible]	[illegible]	[illegible]
40605.004	21820.154	31964.397	30884.861	38600.518	24359.326	36370.276	21138.447	26512.397	19905.447	26697.861	20021.861	26280.861	18956.74

Figure6A（N22T22-N28T28）

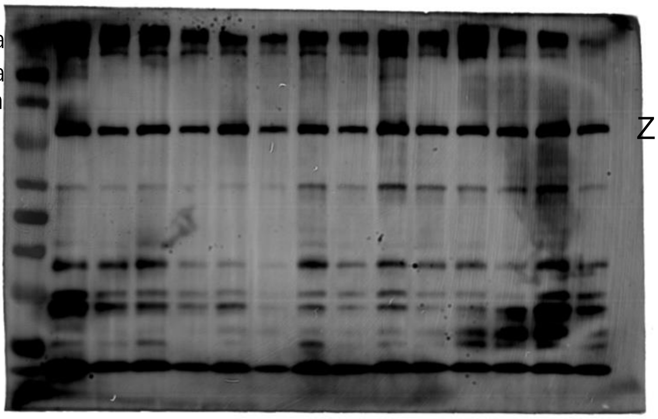

34757.782	14784.983	17396.74	13017.326	25334.861	15840.861	24601.104	16033.447	30404.69	19585.983	21256.104	28460.569	39582.347	16690.69

Figure 6A (N1T1-N7T7)

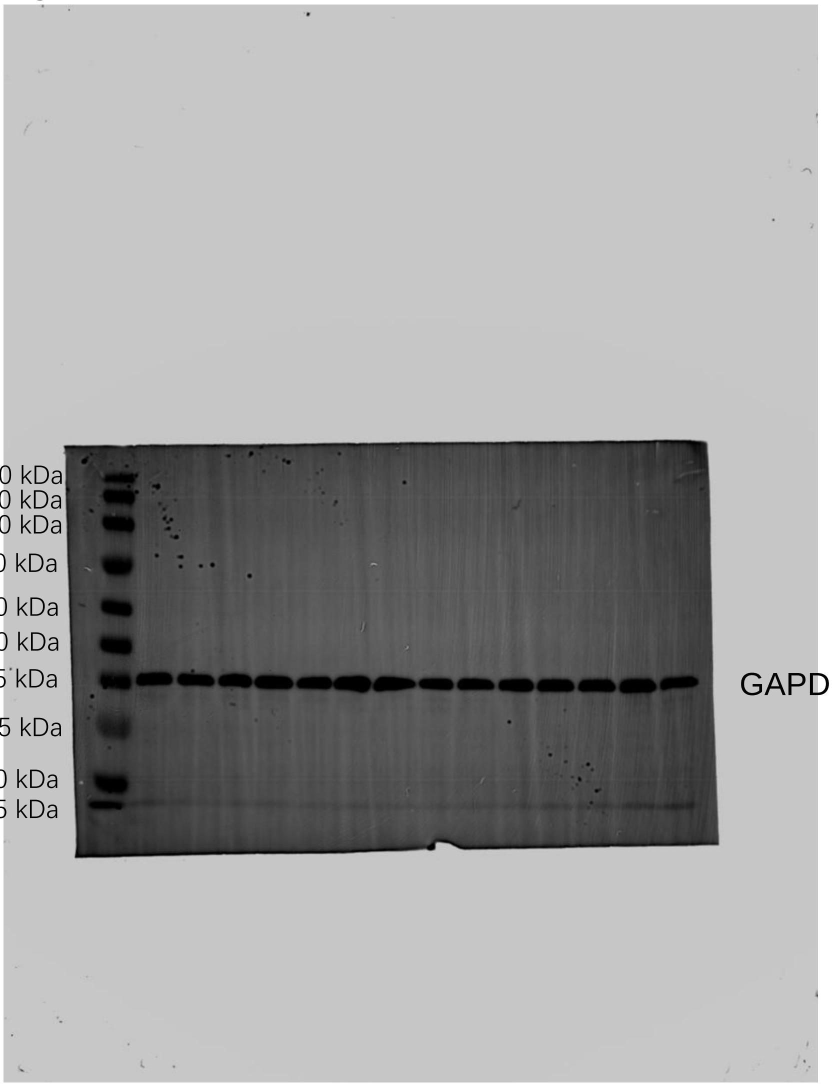

36477.246	38030.276	38221.397	31178.74	32976.74	38398.64	31261.912	31907.447	34824.74	30717.033	38148.811	35755.619	35269.861	31550.69

Figure 6A（N8T8-N14T14）

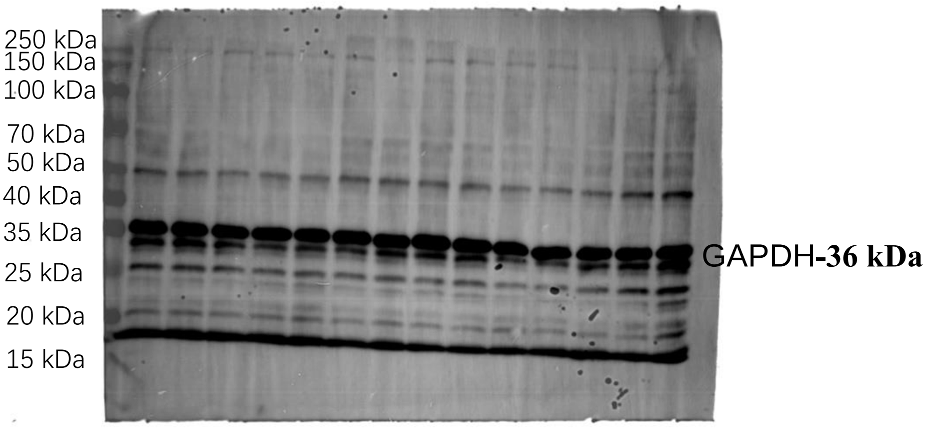

36588.782	37193.74	34697.74	35788.518	34593.154	35980.104	38499.276	39382.962	36326.033	39167.983	38354.104	38788.569	31130.569	37306.246

Figure 6A（N15T15-N21T21）

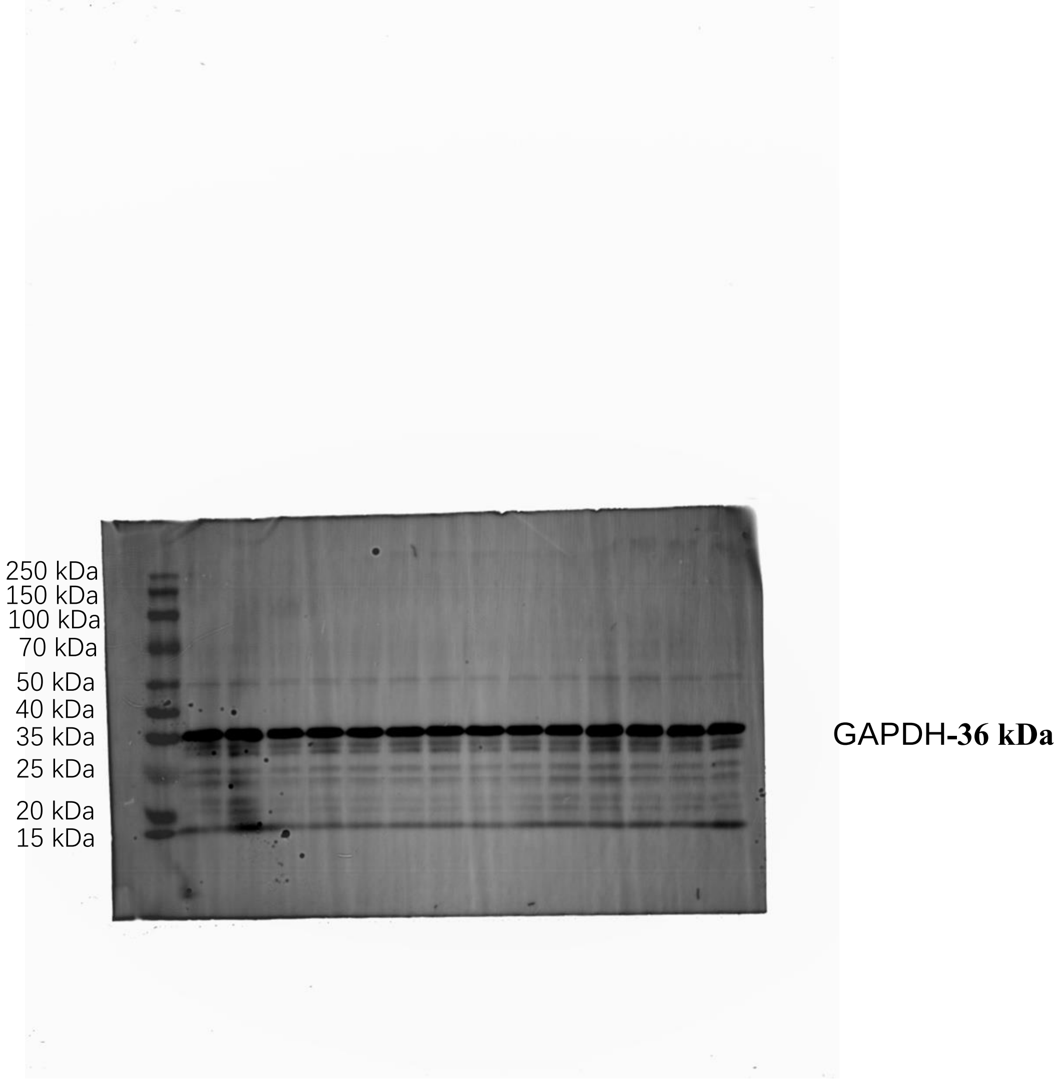

35298.66	36683.276	30248.619	38422.447	39082.033	32877.983	31357.861	38920.497	38400.134	32720.276	38068.912	34743.861	31466.276	38889.811

Figure 6A（N22T22-N28T28）

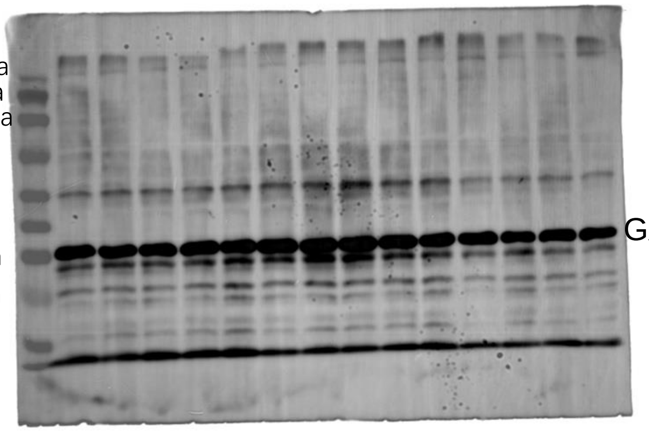

39356.933	38863.619	39691.447	34134.619	39947.79	37355.861	36888.054	38015.569	37622.619	29529.397	29647.841	30752.912	32257.619	35459.154

Figure 6C (ZBTB7C**-69kDa)**

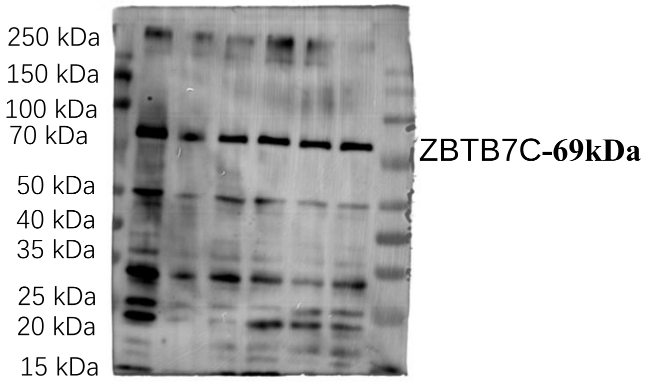

46649.51	24494.095	18011.004	17796.61	17212.196	14240.66

Figure 6C (GAPDH-**36kDa)**

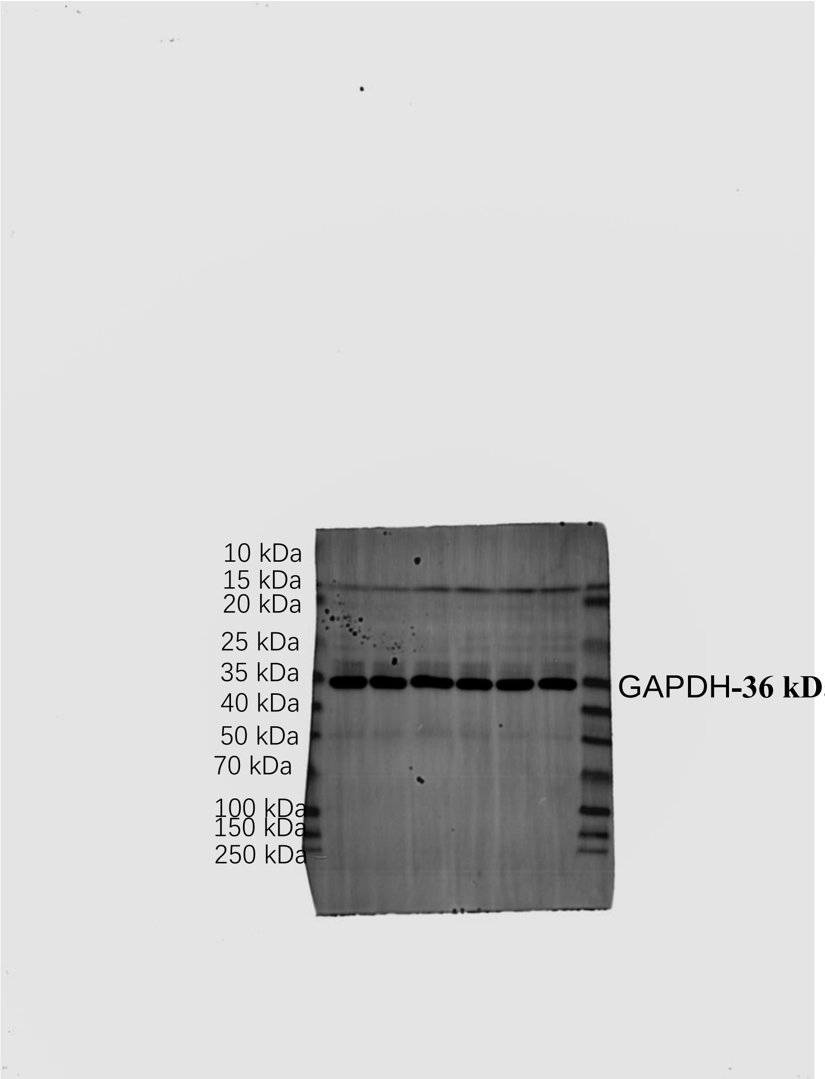

47227.974	47016.903	51720.974	44925.832	43737.468	43874.489

Figure 6E (ZBTB7C**-69kDa)**

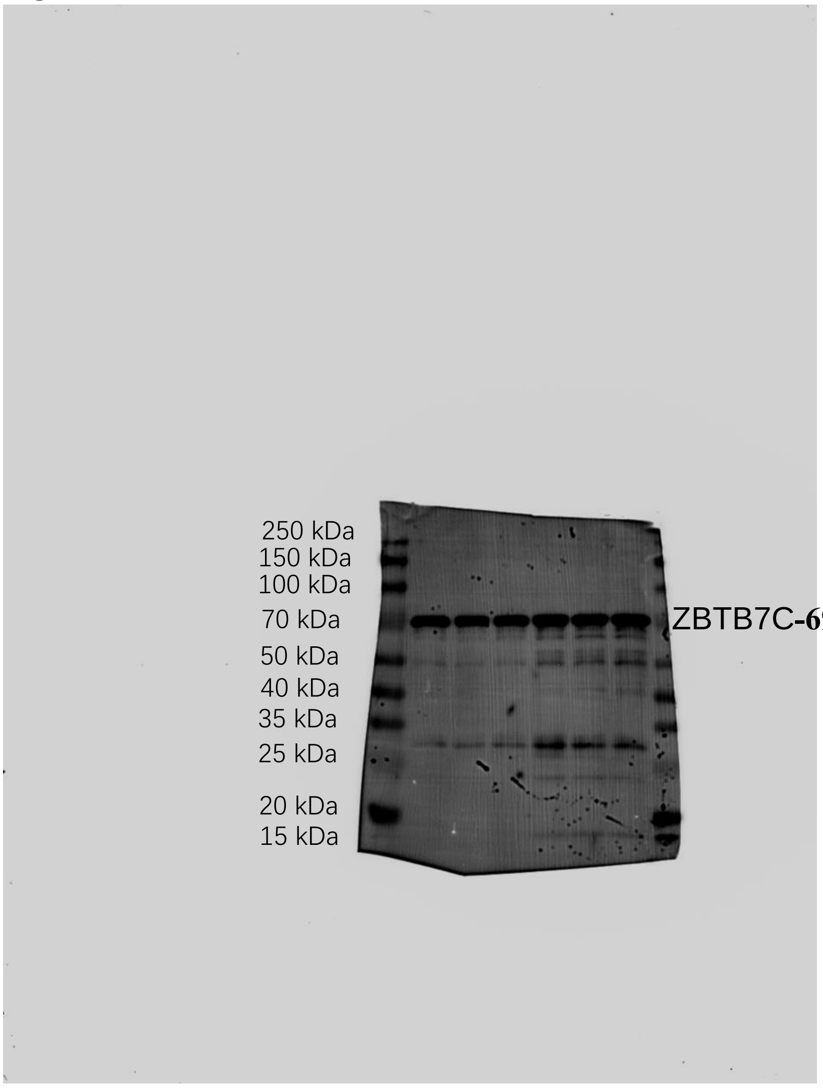

35105.217	30893.539	34354.075	40869.731	39259.439	43706.602

Figure 6E (PCNA-35kDa)

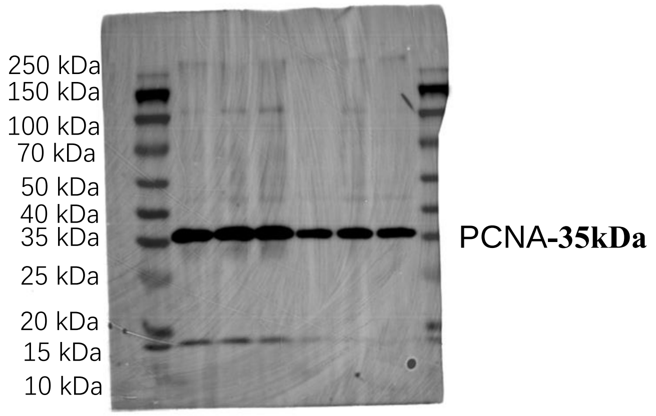

	48535.865	47753.317	47186.731	25995.711	30634.761	26241.61

Figure 6E (GAPDH-36kDa)

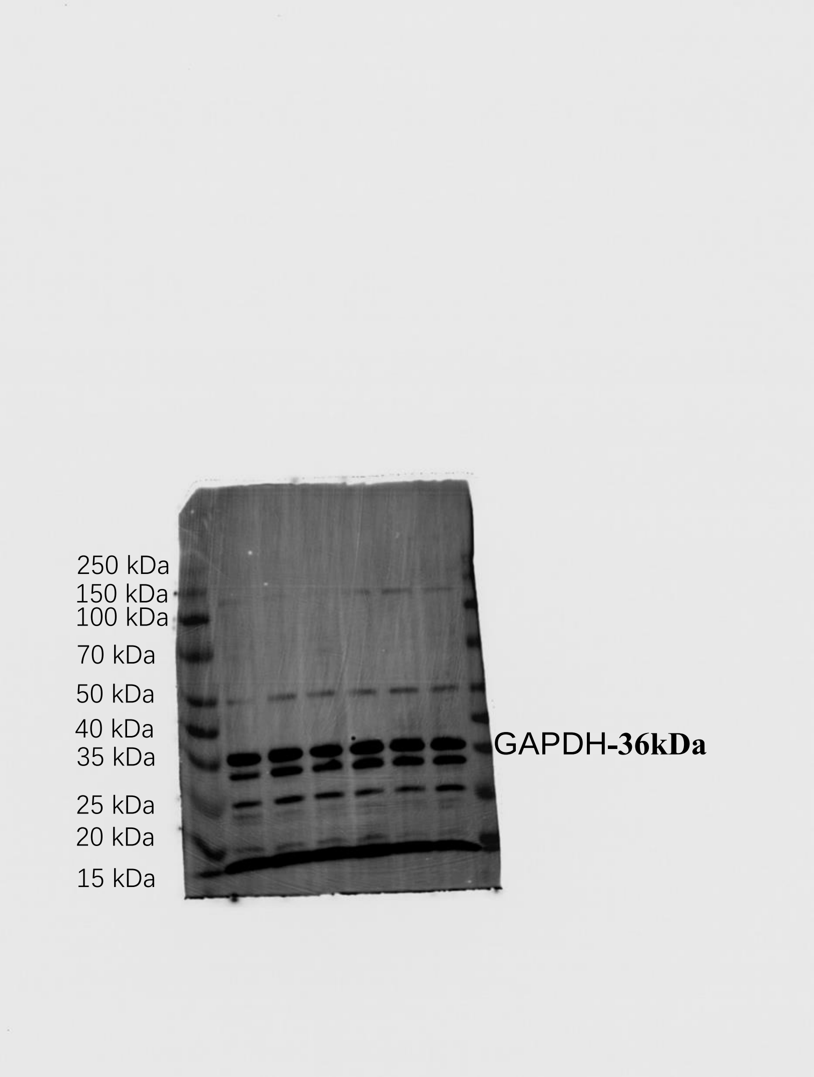

	45581.551	41285.903	45705.51	43236.61	40169.853	36936.459	

Figure 7A (MuRF-1**-42kDa)**

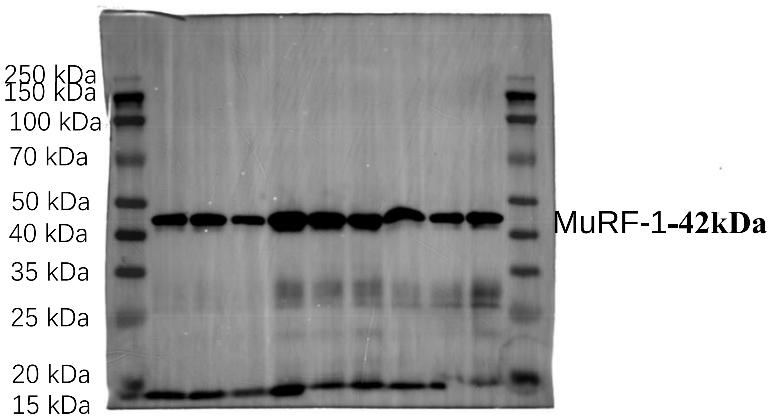

28835.116	29405.125	27430.418	63862.075	65614.004	59119.296	46580.217	41511.125	37638.024

Figure 7A (Atrogin-1-**42kDa)**

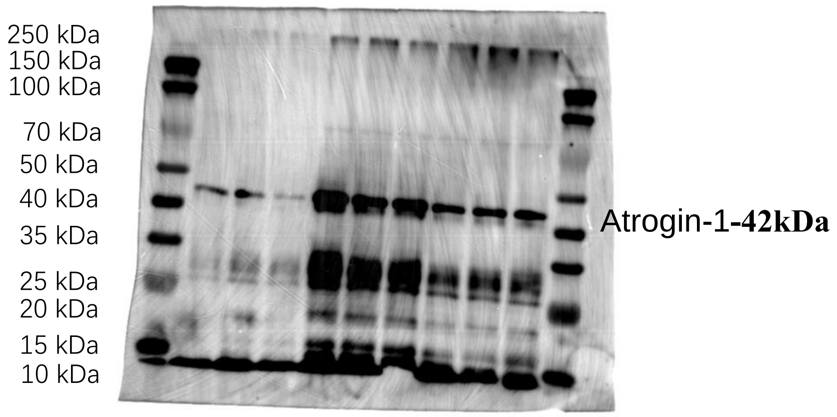

7855.489	10335.075	9512.832	55126.388	62777.267	61663.075	46675.56	42278.924	34098.439

Figure 7A (GAPDH-36**kDa**)

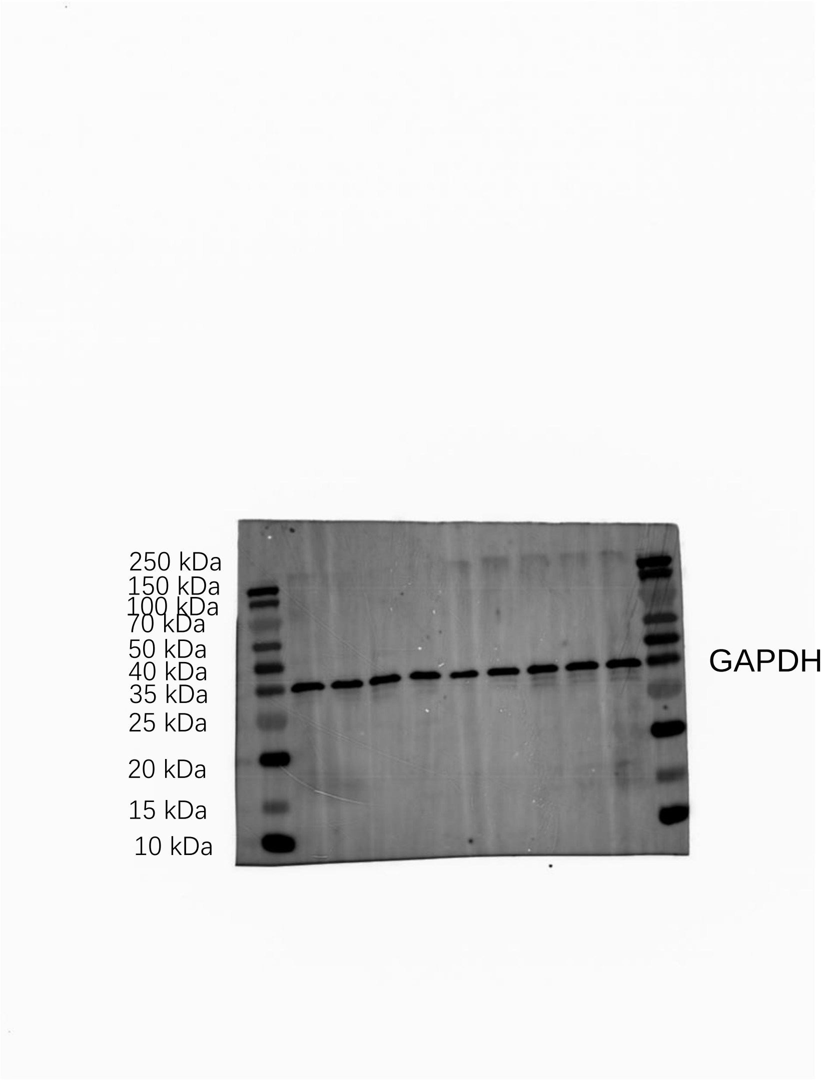

38807.075	36965.439	41355.61	41408.853	40124.075	38009.903	48483.489	45762.974	38652.974

Figure 7A (p-AKT-60kDa)

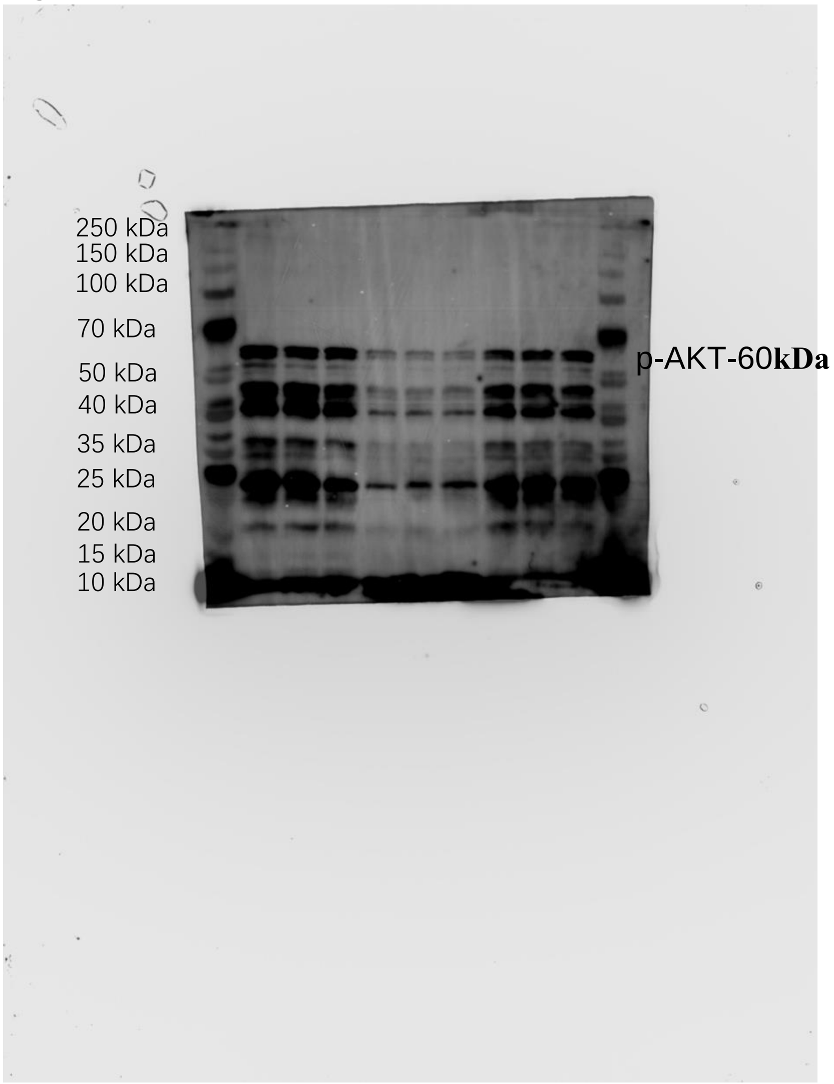

67485.56	49354.974	45503.731	13508.761	11372.518	16440.731	38327.368	41186.196	48139.338

Figure7A(AKT-60kDa)

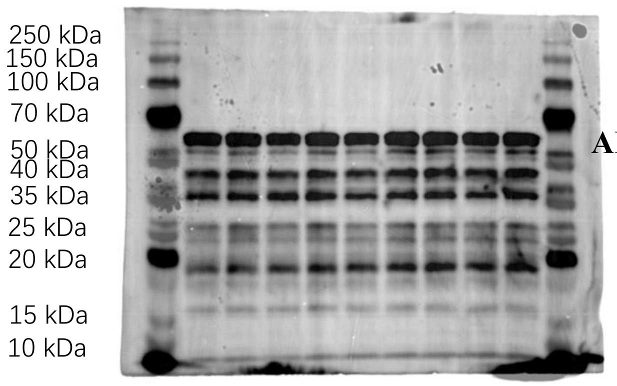

53326.196	50185.024	43591.246	50249.782	45721.731	55662.489	55427.489	53531.368	56306.045

Figure7A(GAPDH-36kDa)

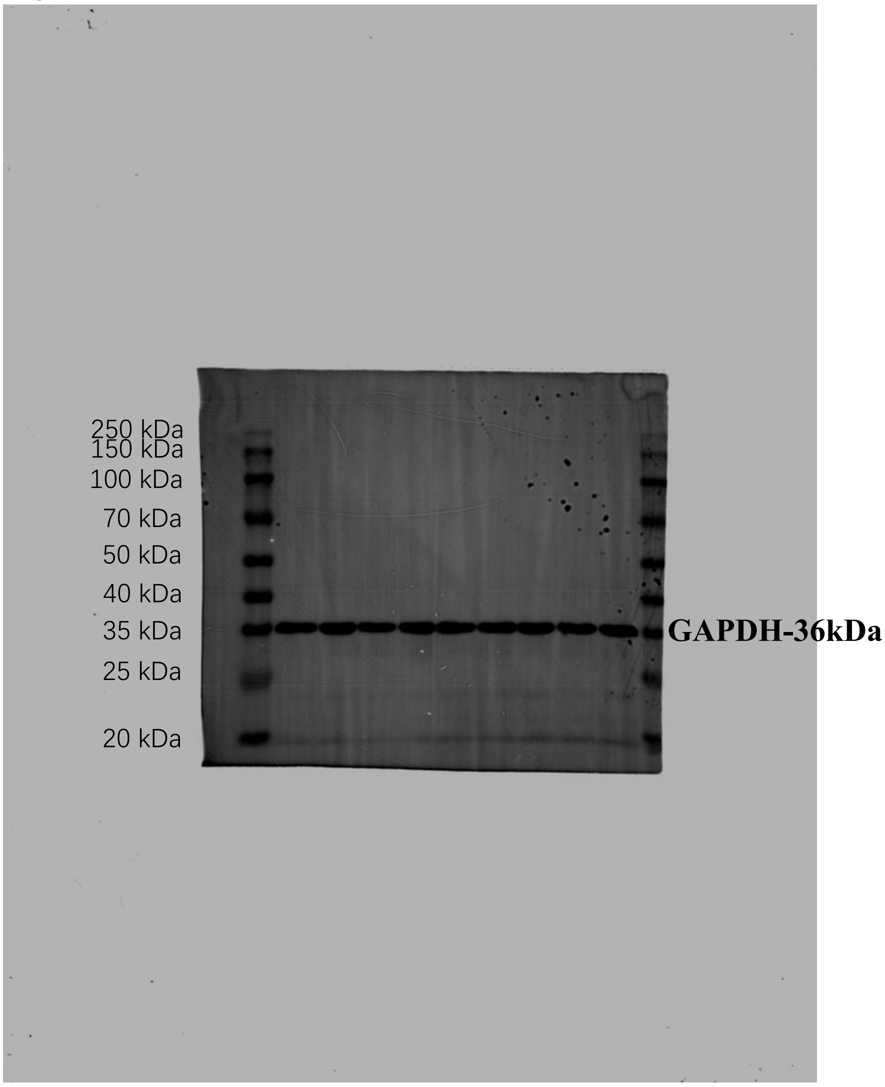

No densitometric quantification was performed for this band.

Figure7A(p-FOXO3A-90kDa)

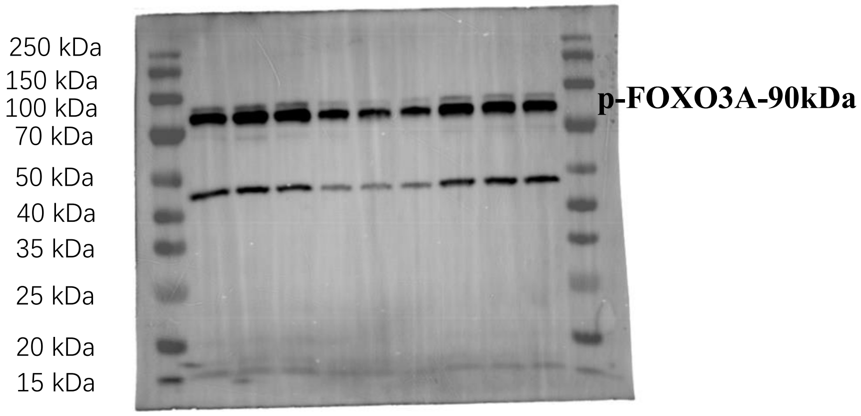

51036.702	60486.731	59393.631	38350.489	33907.51	31740.317	49733.731	45956.246	42375.317

Figure7A(FOXO3A-90kDa)

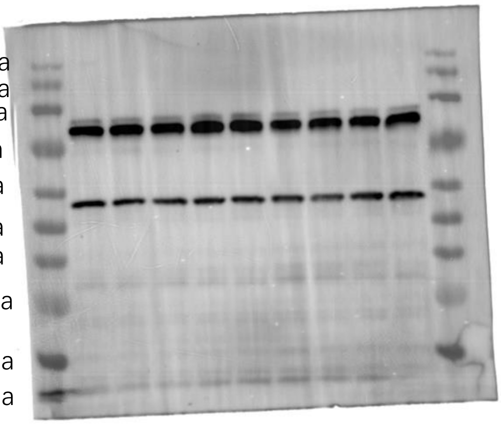

FOXO3A-90kDa

43898.459	48348.782	50245.004	56318.146	54703.146	48014.61	49366.853	45331.317	49603.208

Figure7A(GAPDH-36kDa)

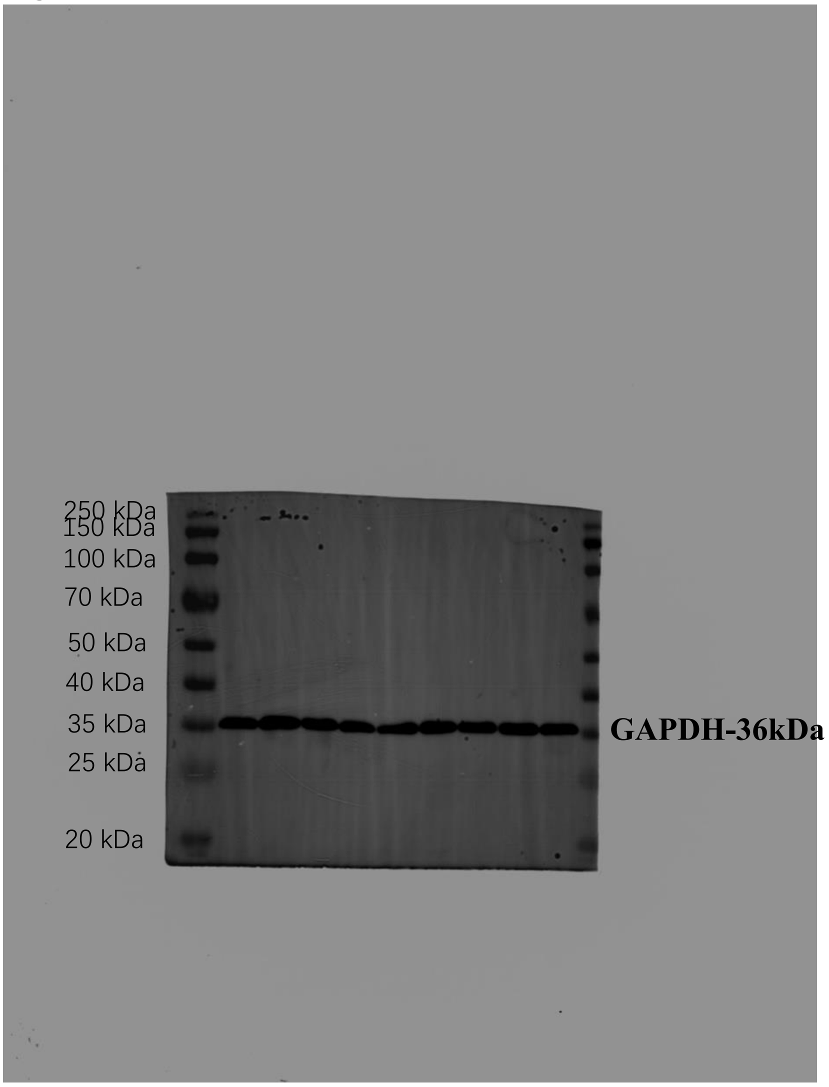

No densitometric quantification was performed for this band.

Figure7A(p-P70S6K-70kDa)

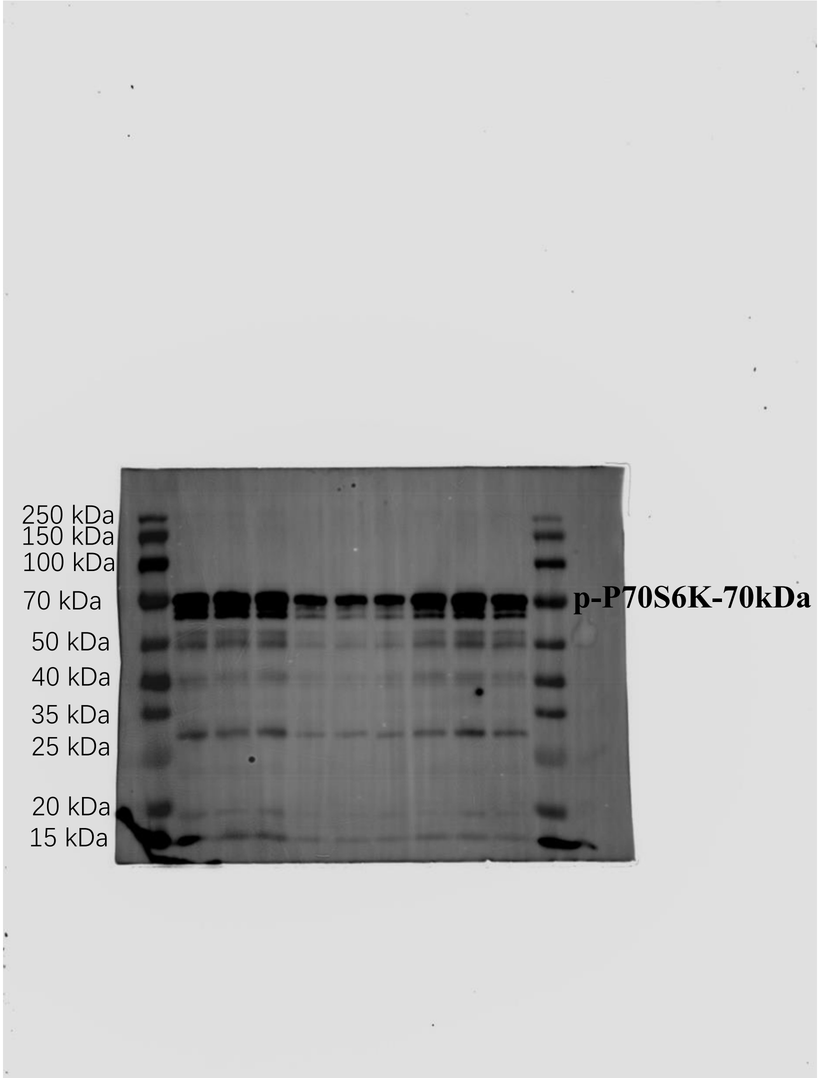

58866.309	63387.974	55698.66	33217.196	29531.489	29504.317	40881.681	46942.51	45617.217

Figure7A(P70S6K-70kDa)

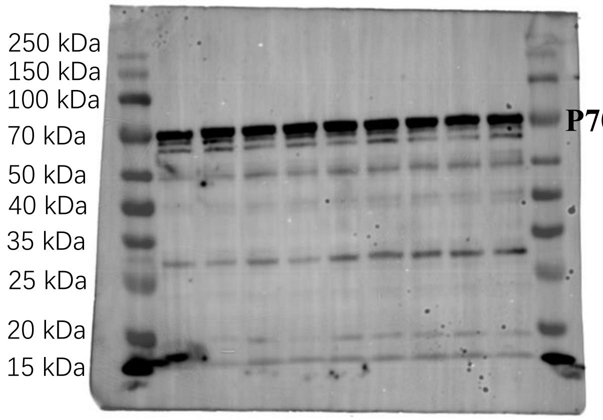

49700.087	51692.56	48027.853	54743.439	52357.782	55963.024	47101.903	45958.924	52631.439

Figure7A(GAPDH-36kDa)

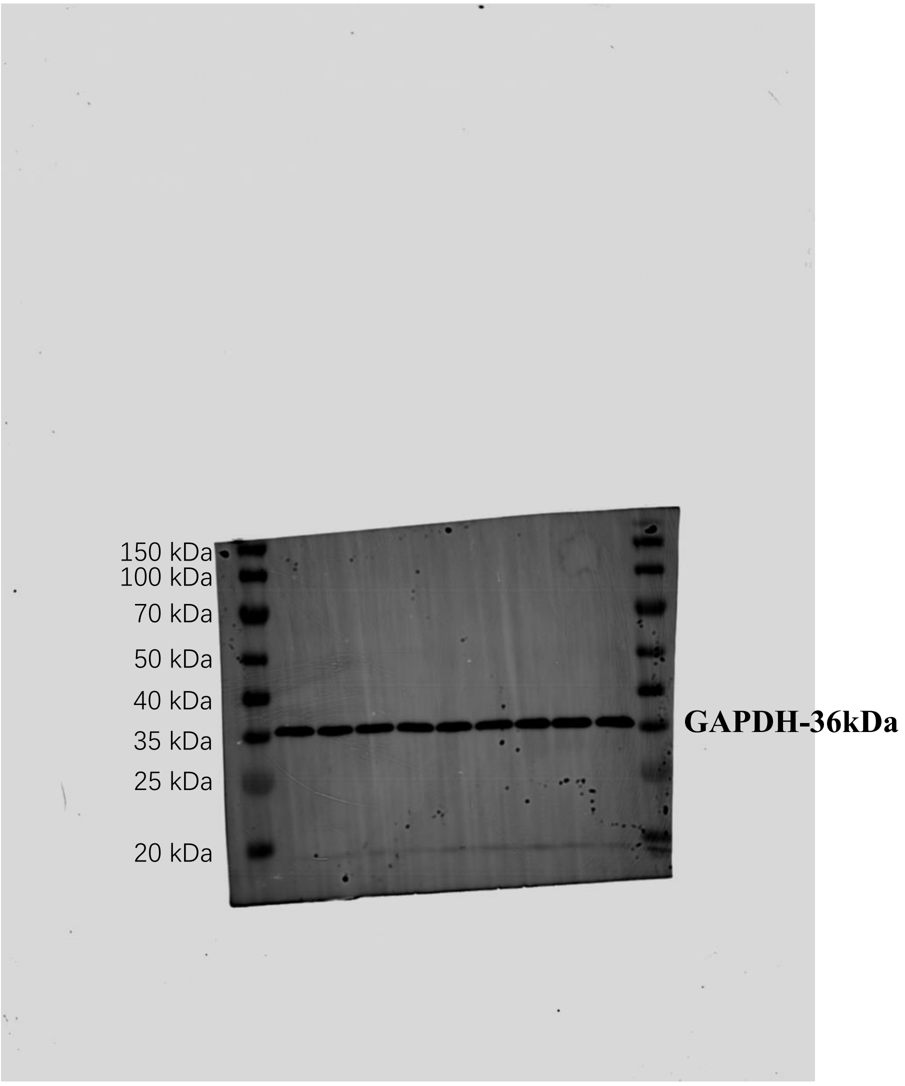

No densitometric quantification was performed for this band.

Figure7A(Puromycin)

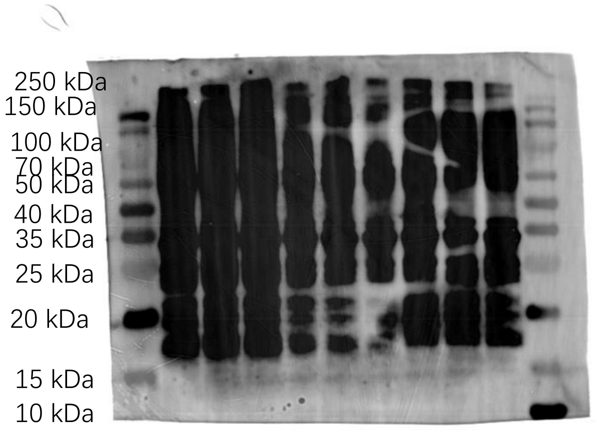

Puromycin

70357.054	70993.64	79699.276	48924.418	49363.581	43349.468	53712.196	53671.317	56734.548

Figure7A(GAPDH-36kDa)

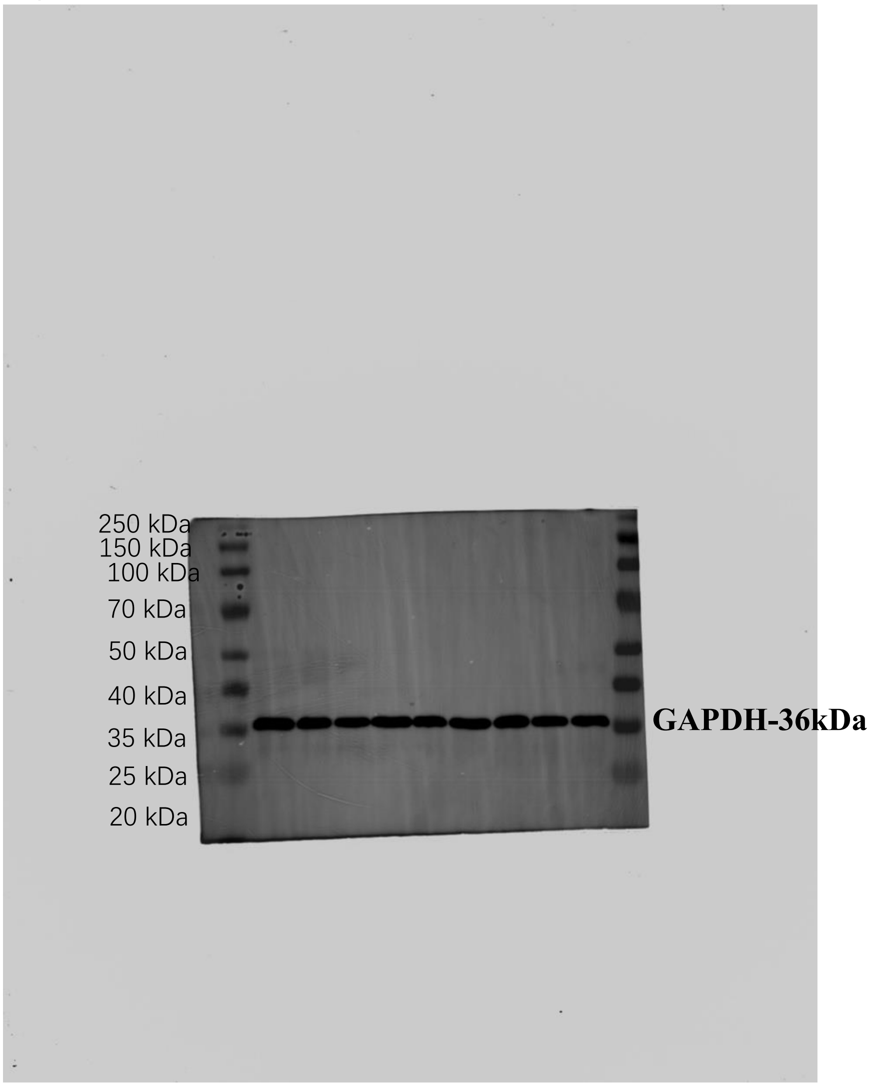

45634.217	43471.711	49639.61	50991.024	52203.489	42708.095	41453.024	43282.953	41690.167	

Figure8H(ZBTB7C-69kDa)

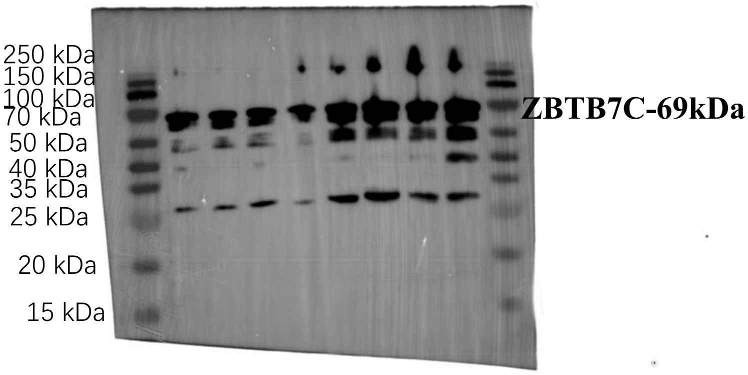

24114.43	20703.903	20331.61	15227.61	33978.267	45205.459	33931.267	45122.116

Figure8H(PCNA-35kDa)

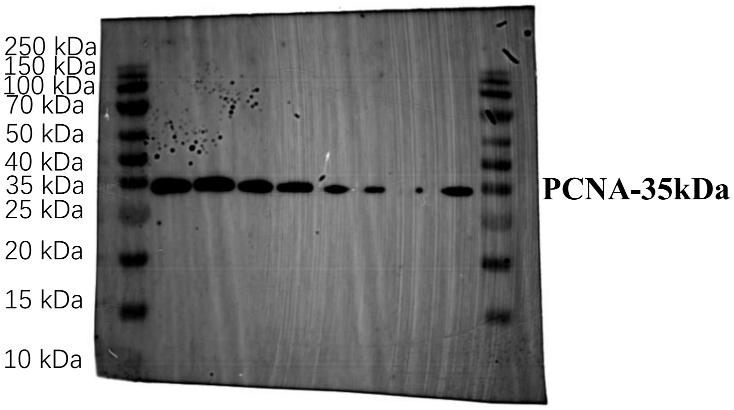

59785.38	59375.045	43965.974	47121.974	18209.267	10886.56	23270.962	24817.803

Figure 8H(GAPDH-36kDa)

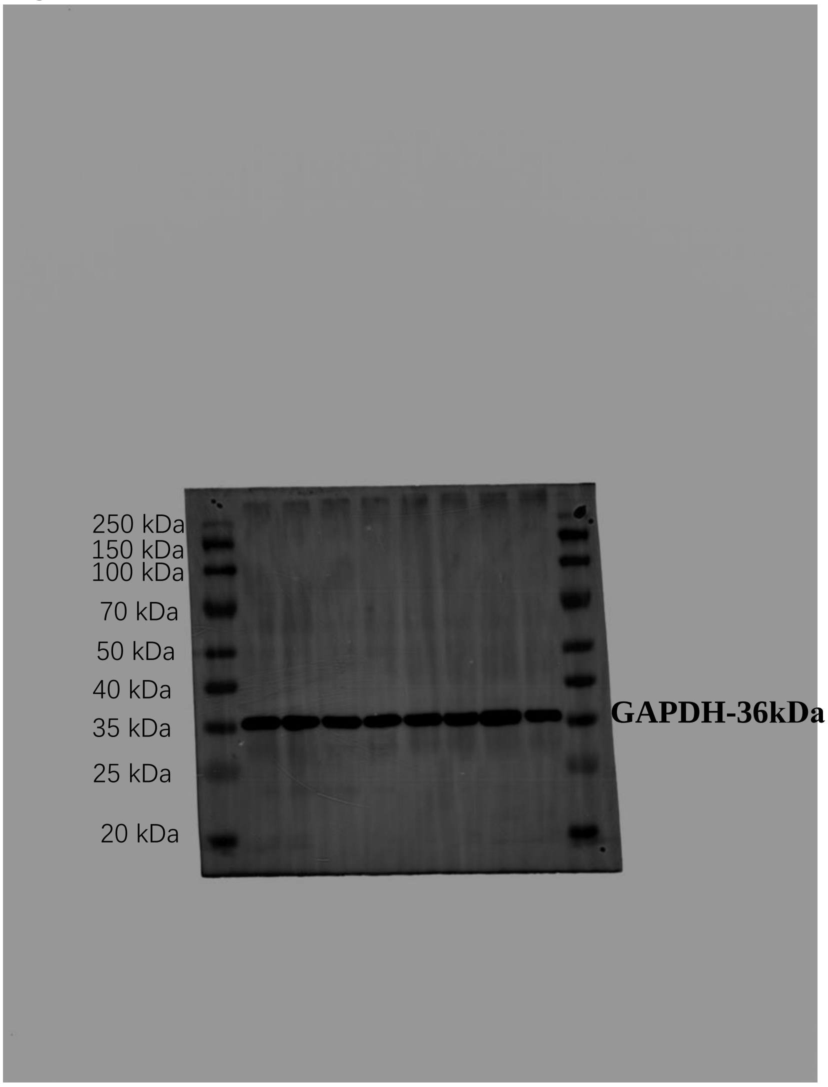

42530.489	49100.924	43108.418	44754.146	40474.832	48432.711	45912.116	44335.631

Figure 9A (MuRF-1**-42kDa)**

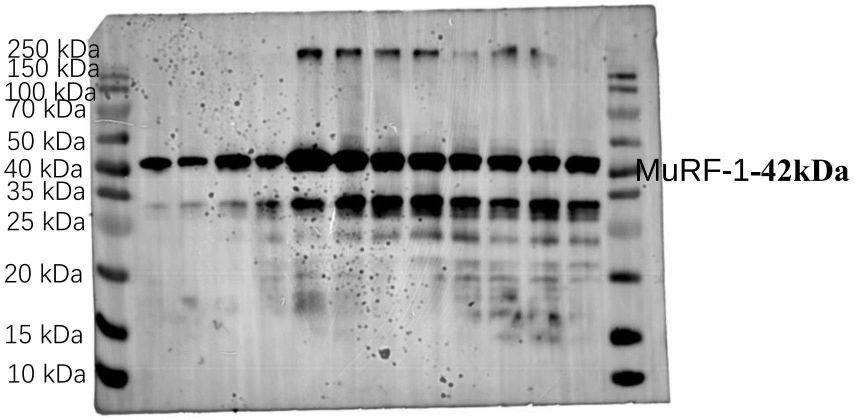

19611.589	16316.518	20296.832	20416.69	46863.66	44663.418	40747.569	42120.953	32257.054	29540.761	26389.468	28722.054

Figure 9A (Atrogin-1-**42kDa)**

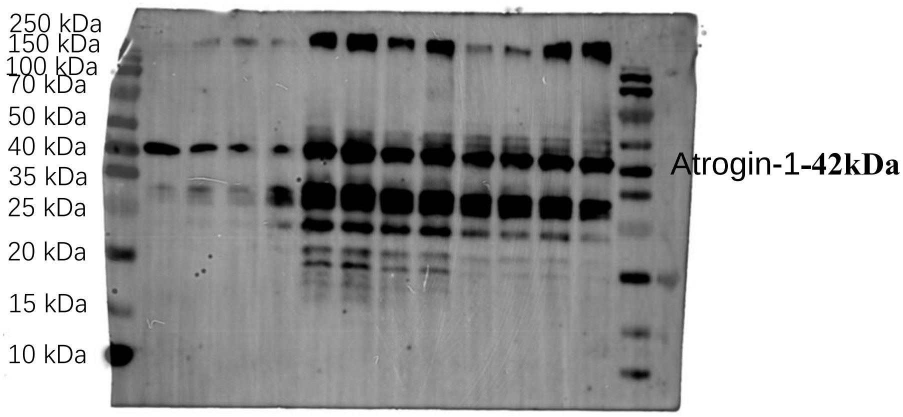

16678.882	11335.64	11186.004	12531.347	49013.953	45279.418	43985.882	43072.004	30863.468	31606.125	33152.368	33248.175

Figure 9A (GAPDH-36**kDa)**

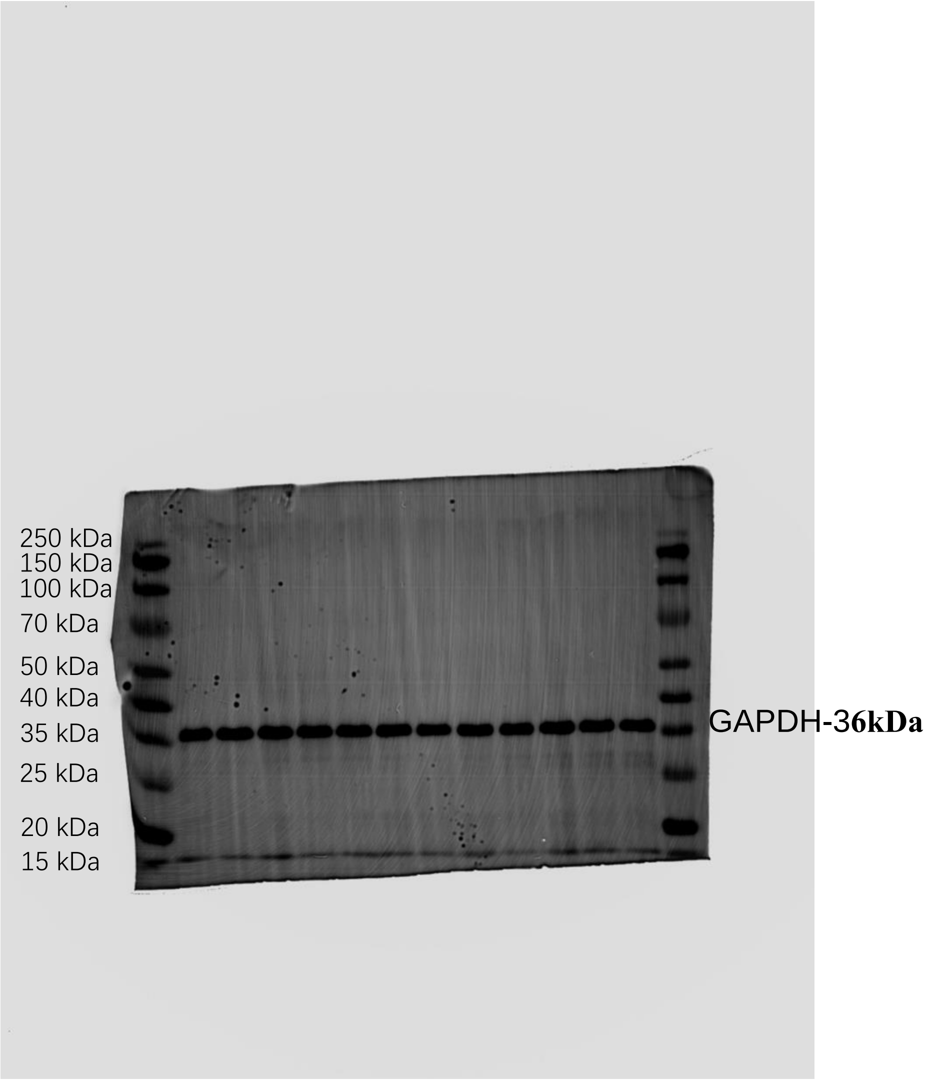

46128.175	41561.953	45616.589	46687.418	48662.711	45232.246	40626.125	44396.903	40955.882	46180.66	40697.368	47739.175

Figure 9A (p-AKT-60**kDa)**

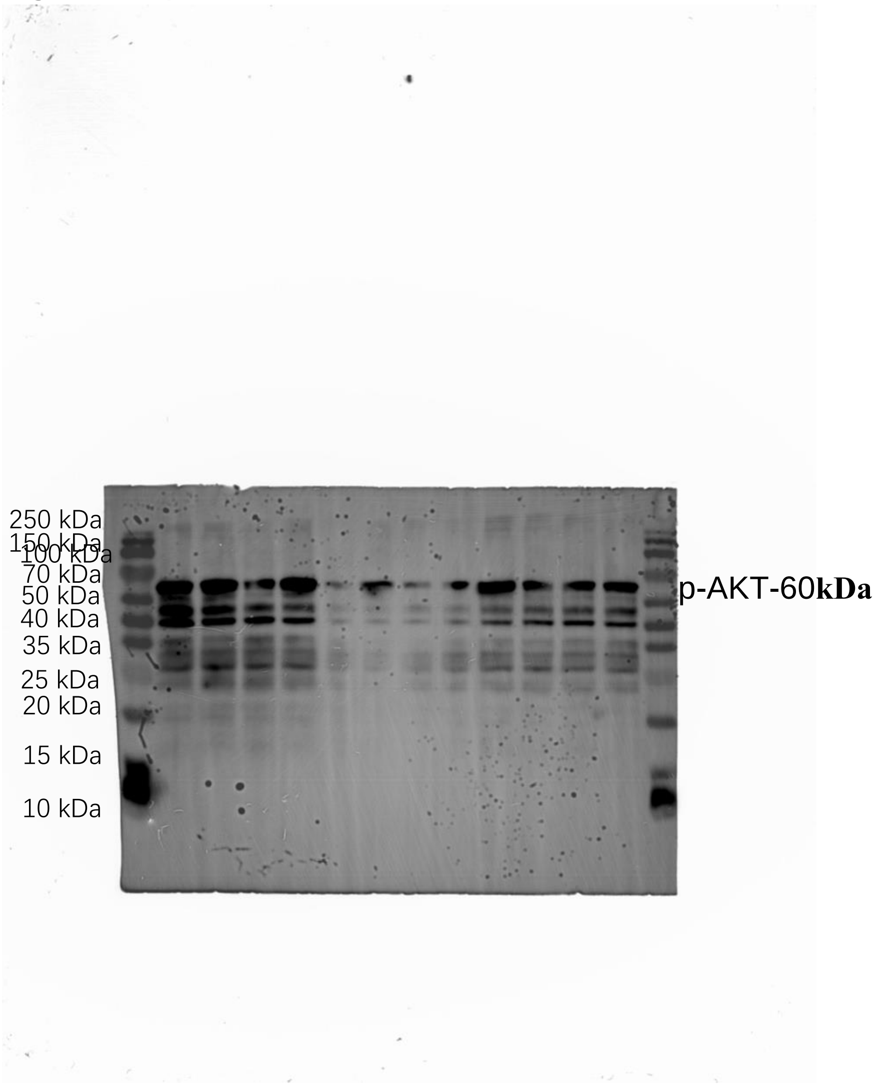

43865.539	44151.468	44281.64	45136.368	7193.447	21307.418	7569.154	13872.397	29327.589	21824.761	23294.64	26596.518

Figure9A(AKT-60kDa)

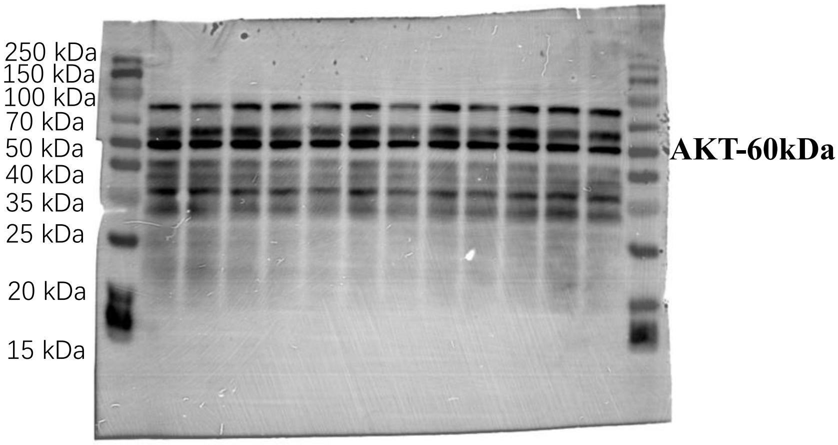

43194.439	42148.711	40703.497	35886.518	34318.104	39262.983	31212.225	36815.225	35443.69	42652.347	36576.761	33486.711

Figure9A(GAPDH-36kDa)

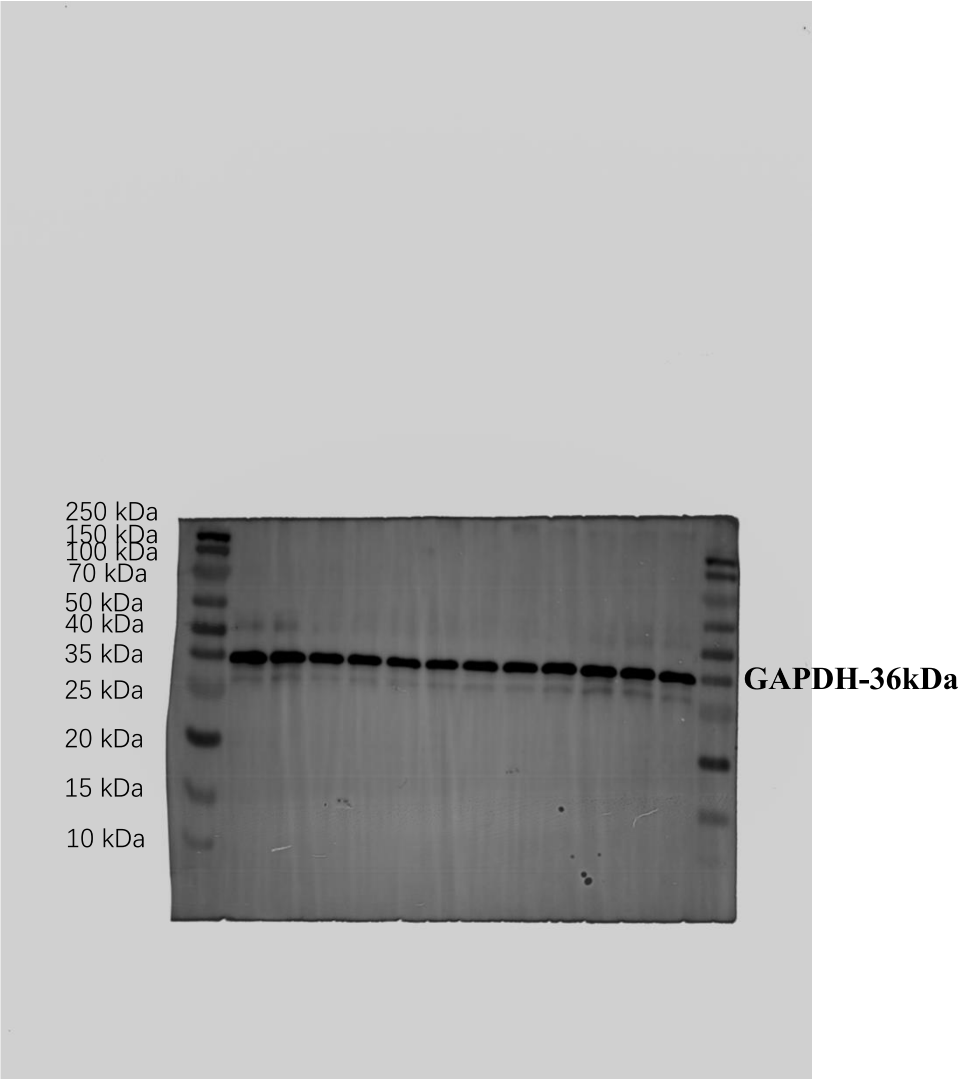

No densitometric quantification was performed for this band.

Figure9A(p-FOXO3A-90kDa)

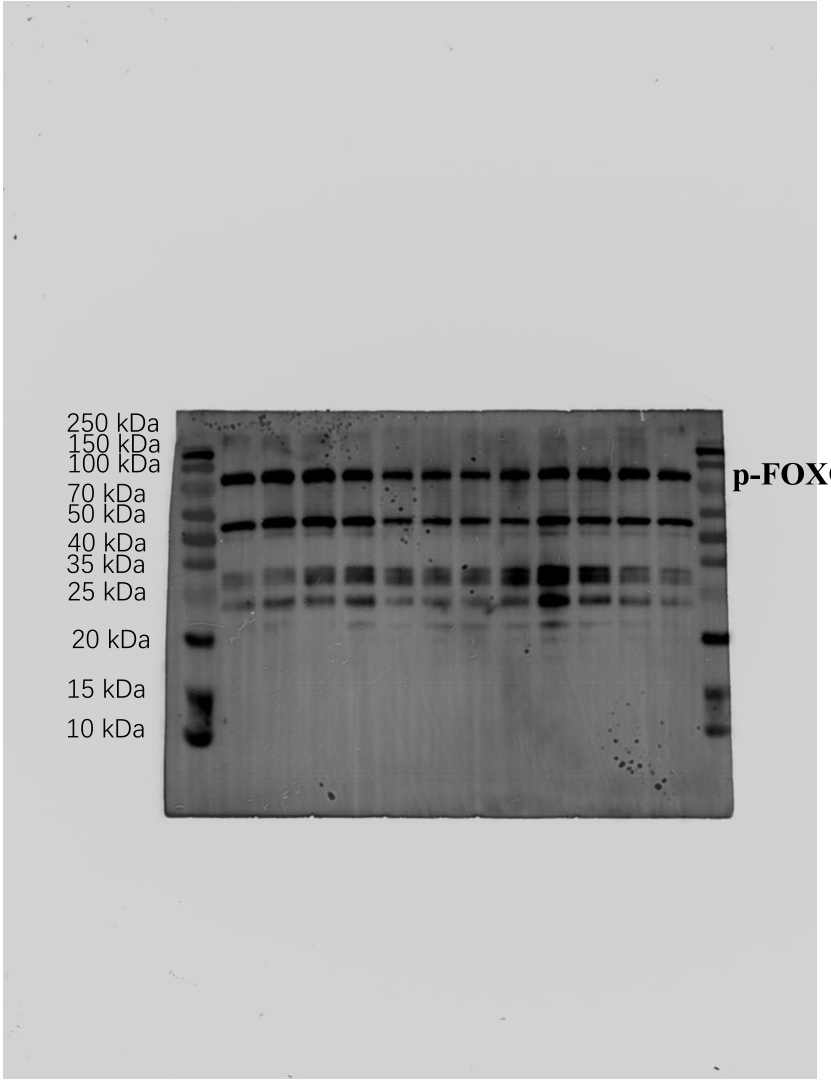

p-FOXO3A-90kDa

40102.589	48311.246	41604.175	45237.811	29845.175	26289.296	23336.125	24935.903	32478.296	35370.175	35122.418	38582.175

Figure9A(FOXO3A-90kDa)

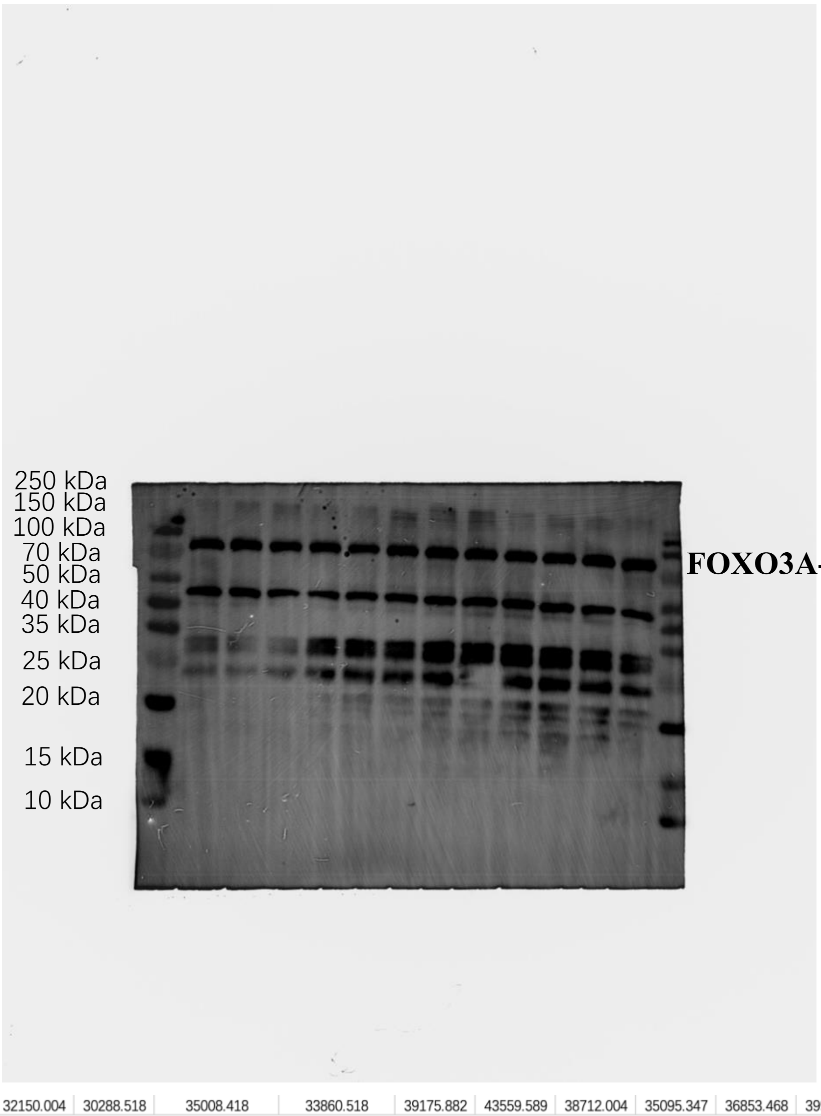

FOXO3A-90kDa

32150.004	30288.518	35008.418	33860.518	39175.882	43559.589	38712.004	35095.347	36853.468	39923.953	36155.589	38651.589

Figure9A(GAPDH-36kDa)

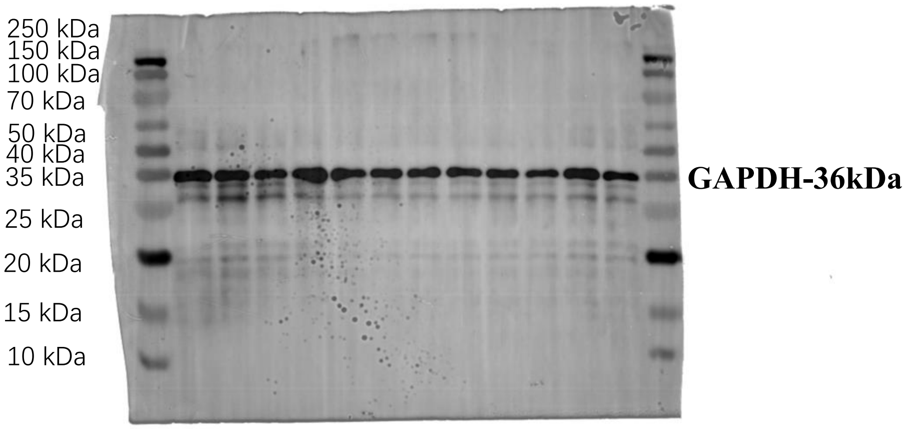

No densitometric quantification was performed for this band.

Figure9A(p-P70S6K-70kDa)

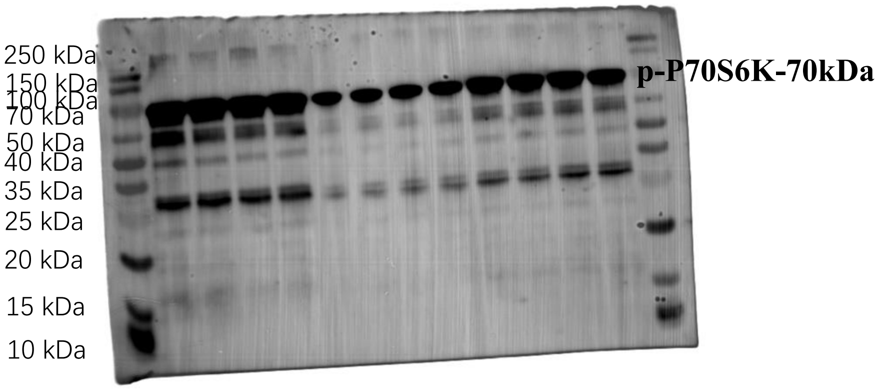

52773.459	45787.296	45196.539	45856.296	21858.104	24278.539	25120.882	28350.539	39424.731	39517.246	37922.539	33436.61

Figure9A(P70S6K-70kDa)

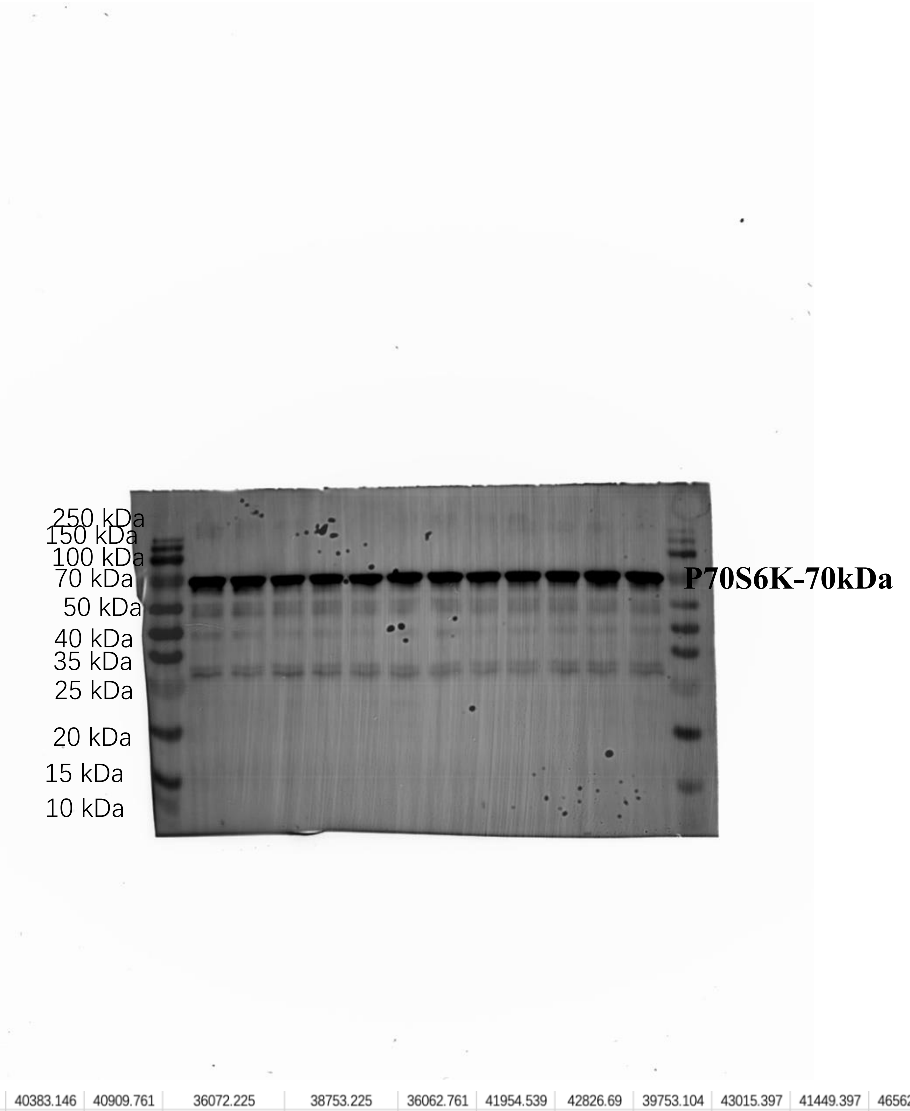

40383.146	40909.761	36072.225	38753.225	36062.761	41954.539	42826.69	39753.104	43015.397	41449.397	46562.296	43473.539

Figure9A(GAPDH-36kDa)

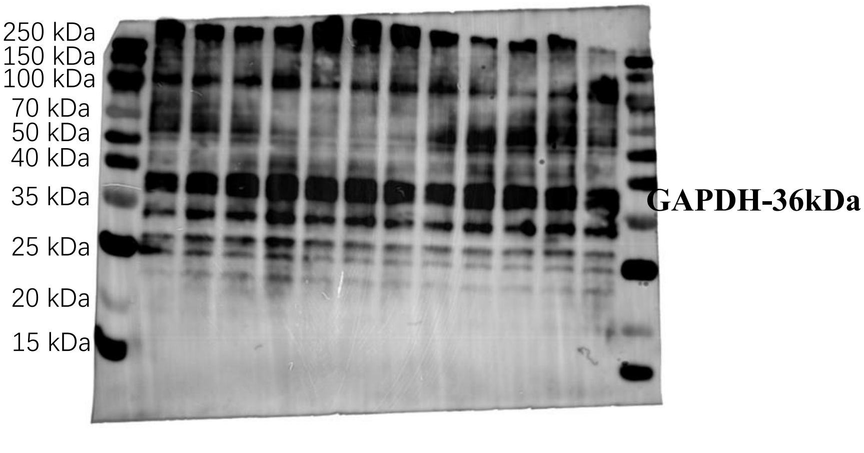

No densitometric quantification was performed for this band.

Figure9A(Puromycin)

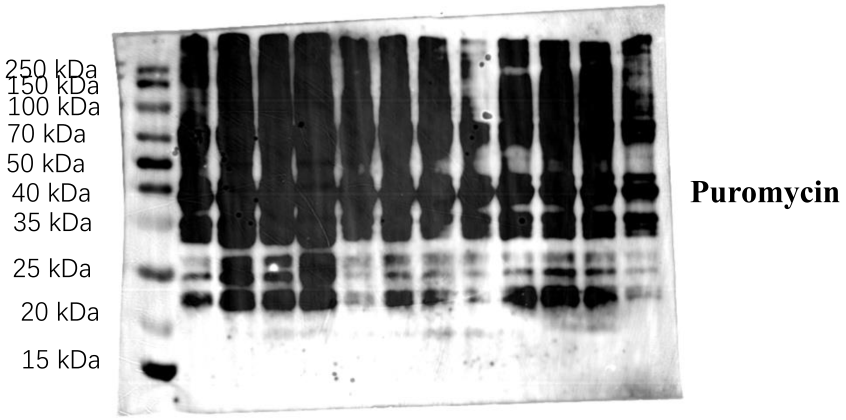

69190.953	75065.527	72819.234	79031.811	22634.113	21318.619	20253.962	18047.326	33809.782	43650.376	43971.376	31754.267

Figure9A(GAPDH-36kDa)

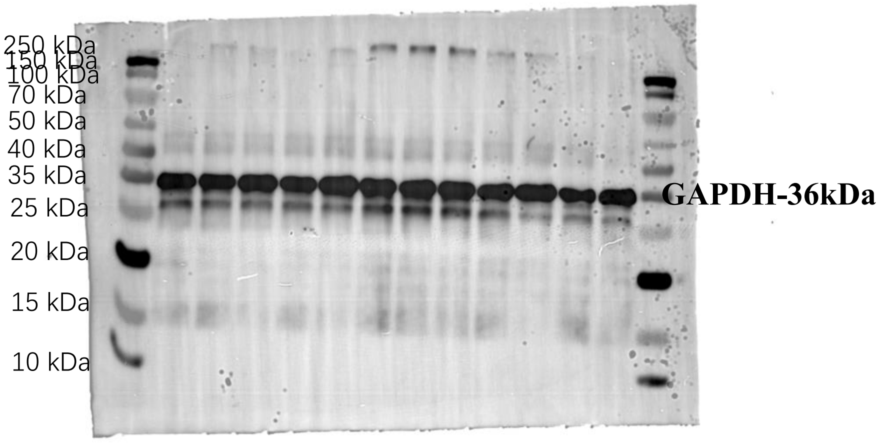

40691.803	35483.64	37365.882	37469.468	40919.125	43954.468	44619.761	40554.418	36052.054	36334.782	40394.711	37847.296
