# Supplementary material for: A Literature-Derived Muscle Wasting-Related Prognostic Signature Identifies ZBTB7C as a Candidate Associated with Tumor-Related Skeletal Muscle Alterations in Esophageal Cancer
Source: Cancers (Basel). 2026 Oct 28;18(21):3479. doi: 10.3390/cancers18213479 (PMC13762034; doi:10.3390/cancers18213479)
Supplement: Supplementary file 1 [file cancers-18-03479-s001.zip › Table S1. Muscle wasting-related genes included in the bioinformatic analysis.pdf]

Supplementary Table S1. Muscle wasting-related genes included in the bioinformatic analysis

Note: The evidence/source column summarizes representative published evidence supporting the inclusion of each gene in the predefined muscle wasting-related gene set. Evidence was derived from cancer cachexia and other established skeletal muscle atrophy models and does not imply that every gene is a direct causal driver of muscle wasting.

Gene	Full name	Evidence/source	Reference
**FBXO32**	F-box protein 32	Canonical atrogene (Atrogin-1/MAFbx) and muscle E3 ubiquitin ligase; consistently induced across fasting, cancer cachexia, diabetes, renal failure, and other atrophic states; promotes ubiquitin-proteasome-mediated muscle protein degradation.	[20]
**TRIM63**	Tripartite motif containing 63	Canonical atrogene (MuRF1) and muscle E3 ubiquitin ligase; strongly induced in multiple catabolic states and widely used as a molecular marker/mediator of skeletal muscle atrophy.	[20]
**TRIM32**	Tripartite motif containing 32	Muscle E3 ubiquitin ligase implicated in atrophy; promotes plakoglobin-PI3K dissociation and suppresses PI3K-AKT-FOXO signaling during muscle wasting.	[21]
**ASB2**	Ankyrin repeat and SOCS box containing 2	Muscle-enriched E3 ubiquitin ligase; ASB2β overexpression is sufficient to induce progressive skeletal muscle atrophy and contractile dysfunction; Asb2 is also increased in cachectic myonuclear populations.	[22; 23]
**FOXO1**	Forkhead box O1	Atrophy-associated transcription factor activated during catabolic stress; participates in induction of proteolytic and metabolic programs, including atrogene and PDK4 expression.	[23; 24]
**FOXO3**	Forkhead box O3	Central transcriptional regulator of muscle atrophy; directly induces FBXO32 and coordinates ubiquitin-proteasome and autophagy-lysosome pathways; constitutively active FOXO3 is sufficient to cause myotube and myofiber atrophy.	[25]
**PDK4**	Pyruvate dehydrogenase kinase 4	FOXO1-responsive metabolic gene induced during energy deprivation; PDK4 is increased in metabolically reprogrammed/cachectic myonuclei and reflects the shift away from glucose oxidation during wasting.	[26]
**MSTN**	Myostatin	Potent negative regulator of skeletal muscle mass; myostatin signaling contributes to cachexia-associated atrophy, and experimental inhibition of myostatin can preserve muscle mass and function.	[27; 28]
**BNIP3**	BCL2 interacting protein 3	Mitophagy/autophagy regulator induced in atrophic muscle; BNIP3 is increased in experimental and human cancer-associated wasting, and targeted downregulation can partially preserve myofiber size and mitochondrial integrity.	[29]
**BNIP3L**	BCL2 interacting protein 3 like	Mitophagy receptor and FOXO-regulated catabolic gene; increased in atrophic skeletal muscle, including insulin-deficient and disuse models, and implicated in mitochondrial quality-control changes during wasting.	[30; 31]
**ATF4**	Activating transcription factor 4	Stress-inducible transcription factor that contributes to skeletal muscle atrophy; ATF4 activity is sufficient to promote fiber atrophy and is required in several stress-related wasting conditions, including fasting and immobilization.	[32]
**STAT3**	Signal transducer and activator of transcription 3	Core inflammatory/cachexia signaling factor downstream of IL-6-family cytokines; STAT3 activation is observed in cachectic muscle and is sufficient and necessary for experimental skeletal muscle wasting.	[33]
**SOCS3**	Suppressor of cytokine signaling 3	Canonical STAT3 target and marker of IL-6/JAK-STAT activation; strongly induced in skeletal muscle in experimental cancer cachexia and associated with inflammatory remodeling of wasting muscle.	[34; 35]
**CEBPD**	CCAAT enhancer binding protein delta	Catabolic transcription factor downstream of inflammatory/STAT3 signaling; participates in induction of myostatin and ubiquitin-proteasome components during muscle wasting.	[35]
**JUNB**	JunB proto-oncogene, AP-1 transcription factor subunit	Muscle mass-maintenance transcription factor; reduced JunB activity accompanies atrophy, whereas JunB overexpression promotes hypertrophy and prevents denervation-associated muscle loss partly by antagonizing FOXO3-dependent atrogene transcription.	[36]
**EDA2R**	Ectodysplasin A2 receptor	Cachexia-responsive receptor strongly upregulated in wasting muscle; EDA2R-NIK signaling directly induces myotube and skeletal muscle atrophy, whereas EDA2R loss protects tumor-bearing mice from muscle wasting.	[37]
**OSMR**	Oncostatin M receptor	Inflammatory receptor linking tumor-derived oncostatin M to skeletal muscle wasting; muscle-specific OSMR deletion protects tumor-bearing mice and reduces the OSM-EDA2R atrophy program.	[37; 38]
**DDIT4**	DNA damage inducible transcript 4	Also known as REDD1; stress-responsive inhibitor of mTORC1. REDD1 is increased in cachectic muscle, and REDD1 deletion attenuates loss of muscle mass and preserves anabolic signaling in tumor-bearing mice.	[39]
**TRIB3**	Tribbles pseudokinase 3	Stress-responsive pseudokinase induced by nutrient deprivation/ER stress; muscle TRIB3 overexpression exacerbates atrophy, whereas knockout attenuates fasting- and age-related muscle loss.	[40; 41]
**DDIT3**	DNA damage inducible transcript 3	Also known as CHOP; ER-stress/UPR transcription factor whose expression and nuclear activity rise in aging- and immobilization-induced skeletal muscle atrophy. Current evidence supports an atrophy-associated, context-dependent role rather than a universal causal effect.	[42]

Gene	Full name	Evidence/source	Reference
**SQSTM1**	Sequestosome 1	Autophagy receptor p62/SQSTM1 involved in proteostasis; altered p62 accumulation and autophagy are documented in sarcopenic/atrophic muscle and interact with TRIB3-dependent remodeling.	[42]
**MYH1**	Myosin heavy chain 1	Fast skeletal myosin heavy-chain isoform and contractile-phenotype marker; sarcomeric/myosin gene repression or isoform remodeling, including MYH1 changes, occurs in cachexia-specific myonuclear programs.	[20]
**MYH2**	Myosin heavy chain 2	Myosin heavy-chain isoform and contractile/fiber-type marker; MYH2 expression is altered or reduced in cancer-cachexia transcriptomic programs and reflects loss/remodeling of the differentiated contractile phenotype.	[20; 43]
**MYH4**	Myosin heavy chain 4	Fast glycolytic myosin heavy-chain isoform; cachexia induces marked remodeling/repression of sarcomeric genes including MYH4 in specific myonuclear programs, consistent with contractile phenotype disruption.	[20]
**MYH7**	Myosin heavy chain 7	Slow/β myosin heavy-chain isoform; altered or reduced MYH7 expression is observed in experimental cancer-cachexia muscle and serves as a marker of fiber-type/contractile remodeling.	[20; 43]
**ACTA1**	Actin alpha 1, skeletal muscle	Major skeletal muscle sarcomeric actin gene; structural-muscle gene expression including ACTA1 is reduced during C26 cancer cachexia, reflecting loss of contractile integrity.	[34]
**CKM**	Creatine kinase, M-type	Muscle energy-transfer enzyme and differentiated muscle marker; CKM is altered in cachexia-associated contractile/metabolic reprogramming and creatine/CKM metabolism is perturbed in experimental cancer cachexia.	[20; 44]
**IGF1**	Insulin like growth factor 1	Major anabolic regulator of skeletal muscle protein synthesis and growth; loss of muscle IGF1 is associated with reduced muscle mass/strength and can exacerbate consequences of disuse atrophy.	[45]
**PPARGC1A**	PPARG coactivator 1 alpha	PGC-1α coordinates oxidative metabolism and opposes catabolic transcription; PPARGC1A expression decreases in denervation, cancer cachexia, diabetes, and renal-failure atrophy, while overexpression suppresses FOXO3-dependent atrogenes and protects against wasting.	[46]
**IRS1**	Insulin receptor substrate 1	Key insulin/IGF signaling adaptor upstream of PI3K-AKT; IRS1 loss/degradation suppresses anabolic signaling and promotes muscle atrophy, including experimental cancer cachexia.	[47; 48]
**PAX7**	Paired box 7	Muscle satellite-cell self-renewal factor; persistent NF-κB-dependent PAX7 expression in cancer cachexia blocks myogenic differentiation and contributes to muscle wasting; reducing PAX7 restores differentiation and attenuates wasting.	[49]
**FHL1**	Four and a half LIM domains 1	Skeletal muscle structural/signaling protein involved in myostatin pathway modulation; experimental FHL1 can enhance myostatin-dependent wasting, indicating a context-dependent link to muscle atrophy.	[50]
