# Supplementary material for: A Literature-Derived Muscle Wasting-Related Prognostic Signature Identifies ZBTB7C as a Candidate Associated with Tumor-Related Skeletal Muscle Alterations in Esophageal Cancer
Source: Cancers (Basel). 2026 Oct 28;18(21):3479. doi: 10.3390/cancers18213479 (PMC13762034; doi:10.3390/cancers18213479)
Supplement: Supplementary file 1 [file cancers-18-03479-s001.zip › Table S2. Antibodies used for Western blotting.pdf]

Supplementary Table S2. Antibodies used for Western blotting

Antibody	Host/Clonality	Working Dilution	Manufacturer/Location	Catalog No.
MuRF-1	rabbit polyclonal	1:1000	Proteintech, Rosemont, IL, USA	55456-1-AP
Atrogin-1	rabbit polyclonal	1:1000	Proteintech, Rosemont, IL, USA	12866-1-AP
p-AKT	rabbit polyclonal	1:1000	Proteintech, Rosemont, IL, USA	28731-1-AP
AKT	rabbit polyclonal	1:1000	Proteintech, Rosemont, IL, USA	10176-2-AP
p-FOXO3A	rabbit polyclonal	1:1000	Proteintech, Rosemont, IL, USA	28755-1-AP
FOXO3A	rabbit polyclonal	1:1000	Proteintech, Rosemont, IL, USA	10849-1-AP
p-P70S6K	rabbit recombinant monoclonal	1:1000	Proteintech, Rosemont, IL, USA	82373-1-RR
P70S6K	rabbit polyclonal	1:1000	Proteintech, Rosemont, IL, USA	14485-1-AP
ZBTB7C	mouse monoclonal	1:1000	antibodies-online GmbH, Aachen, Germany	ABIN8067707
PCNA	rabbit polyclonal	1:1000	Proteintech, Rosemont, IL, USA	10205-2-AP
Puromycin	rabbit monoclonal	1:1000	ABclonal, Wuhan, China	A23031
GAPDH	mouse monoclonal	1:10000	Proteintech, Rosemont, IL, USA	60004-1-Ig
HRP-conjugated goat anti-rabbit IgG (H+L)	goat polyclonal	1:10000	Proteintech, Rosemont, IL, USA	SA00001-2
HRP-conjugated goat anti-mouse IgG (H+L)	goat polyclonal	1:10000	Proteintech, Rosemont, IL, USA	SA00001-1
